# Post-hemorrhagic hydrocephalus of prematurity is associated with disruption of tight junctions and increased macrophage activity in the choroid plexus

**DOI:** 10.1186/s12987-026-00800-x

**Published:** 2026-03-31

**Authors:** Maria Garcia-Bonilla, Rajiv Swarup, Owen W. Limbrick, Habeebah Z. Vohra, Ayodamola Otun, Konrad McKalip, William Bernhardt, Kirill Shumilov, Marie Michenkova, Jayne Crouthamel, Mackenzie Newman, Krikor Dikranian, James P. McAllister II, David D. Limbrick Jr

**Affiliations:** 1 Department of Neurosurgery, Virginia Commonwealth University School of Medicine, 417 North 11th Street, Sixth Floor, Richmond, VA 23298 USA; 2 Department of Neurosurgery, Washington University in St. Louis School of Medicine, 660 South Euclid Avenue, St. Louis, MO 63110 USA; 3 Office of the Vice President for Research and Innovation. Bioimaging and Applied Research Core, Virginia Commonwealth University, 800 East Leigh Street, Richmond, VA 23298 USA; 4 Department of Neuroscience, Washington University in St. Louis School of Medicine, 660 South Euclid Avenue, St. Louis, MO 63110 USA

**Keywords:** Post-hemorrhagic hydrocephalus, Inflammation, Choroid plexus, Border-associated macrophages, Tight junctions

## Abstract

**Background:**

Previous studies on intraventricular hemorrhage (IVH), a common and severe complication of preterm birth, and subsequent post-hemorrhagic hydrocephalus (PHH), have predominantly concentrated on the secretory function of the choroid plexus (ChP), with considerably less emphasis on its barrier function. We hypothesized that PHH is associated with immune-related alterations in the junction biology of ChP.

**Methods:**

We examined differences in tight junctions and macrophages using a neonatal mouse model of PHH (*n* = 40) and in vitro ChP explants (*n* = 22), as well as human post-mortem samples (*n* = 6). To test our hypothesis, we employed histology, immunofluorescence, magnetic resonance imaging, spectral flow cytometry, fluorescence activated cell sorting, and transmission electron microscopy.

**Results:**

In the mouse model, we observed a significant increase (*p* = 0.0025) in ventricular volume in the PHH group compared to sham controls. PHH was associated with a significant increase (*p* = 0.0177) in the number of macrophages in the ChP. These macrophages displayed an activated phenotype, characterized by numerous phagosomes and lysosomes observed by transmission electron microscopy, and quantified by CD68 immunostaining (*p* = 0.0003). Further, we identified significant decreases (*p* = 0.0048 and *p* = 0.033, respectively) in tight junction proteins ZO-1 and claudin-1 in the epithelial cells of the ChP in PHH. In vitro co-cultures of peripheral CD11b^+^ Ly6G^−^ Ly6C^+^ cells (precursors of ChP macrophages) and lysed blood demonstrated significant disruption (*p* = 0.0046) of tight junctions in ChP. This disruption in ZO-1 was not observed when ChP were cultured only with lysed blood and without CD11b^+^ Ly6G^−^ Ly6C^+^ cells. The findings of tight junction disruption in the ChP epithelial cells and the significant increase (*p*≤0.05) in macrophages were confirmed in preterm human post-mortem ChP samples.

**Conclusions:**

These results suggest that IVH/PHH is associated with an increased in activated macrophages in the ChP and impaired tight junctions in the ChP epithelium. This research opens avenues for exploring novel immunomodulatory treatments aimed at preventing the pathogenesis and neurodevelopment impairments common in PHH.

**Supplementary Information:**

The online version contains supplementary material available at 10.1186/s12987-026-00800-x.

## Background

The choroid plexus (ChP) is a secretory tissue responsible for producing cerebrospinal fluid (CSF) in the vertebrate brain and is composed of an epithelial monolayer resting on a basal lamina and stroma containing fenestrated blood vessels [1, 2]. The ChP also constitutes the blood-CSF barrier, regulating the trafficking of immune cells between the CSF and blood in response to infection or tissue damage [3–11]. Its position allows it to detect changes and trigger appropriate responses, which is crucial for maintaining homeostasis but can lead to dysregulation in disease [4, 12].

Intraventricular hemorrhage (IVH) is the most frequent, severe complication of preterm birth [13–19]. High grade IVH results in post-hemorrhagic hydrocephalus (PHH) in 40–50% of the cases and is associated with disordered homeostasis of the ChP-CSF system and inflammation [20]. Treatment of PHH involves the placement of a ventriculoperitoneal shunt to divert CSF from the ventricles to the peritoneal cavity for abosrption [21–23]. However, the failure rate of ventriculoperitoneal shunting is high, between 11% and 25% in the first year and > 90% after ten years [20, 24–27]. The lack of long-term surgical interventions and poor understanding of PHH pathophysiology underscore the need to study the ChP pathophysiology of PHH to ultimately design new therapeutic approaches.

Key constituents of the blood-ChP-CSF barrier are the epithelial cells, linked by tight junction proteins [28–31]. Furthermore, the ChP has long been recognized for its role in neuroimmune functions [32–34]. There are macrophages located in border regions, such as the meninges and ChP [35]. Macrophages are composed of various subpopulations in the ChP, including those derived from peripheral CD11b^+^ Ly6G^−^ Ly6C^+^ cells (monocytes) [3, 36–38]. These ChP macrophages that express several macrophage markers including CD45, CD11b, F4/80, CD206, and Iba1 [36]. All are considered important players in neuroinflammation, development, tissue repair, and immunological memory [39, 40]. Research on other brain diseases have shown that proteases secreted by ChP macrophages could impair the blood-CSF barrier [41]. However, the contribution of macrophages to ChP tight junction integrity in the context of IVH/PHH remain to be elucidated. We hypothesized that IVH/PHH is associated with immune response of macrophages and ChP tight junction disruption. We compared tight junction integrity and immune cell populations between PHH and control groups via flow cytometry, immunofluorescence, and transmission electron microscopy (TEM).

## Methods

### Autopsy brain specimens

Post-mortem ChP samples were obtained from human preterm infants born at 25 ± 2.4 post-menstrual age diagnosed either with IVH/PHH (*n* = 3) or no intracranial hemorrhage, infection, or hydrocephalus (control, *n* = 3, Suppl Table 1). Group classification was based on clinical ultrasound evaluation. The human subjects research committees of all institutions waived or approved all procedures (IRBs #201101887 Legacy #09-0183, and #201203126).

### Experimental animals

C57BL/6 mice (*Mus musculus*) were obtained from The Jackson Laboratory (Bar Harbor, ME, USA) and Charles River (Wilmington, MA, USA). They were bred in the Washington University School of Medicine and Virginia Commonwealth University at 22 °C with a 12:12 light/dark cycle and standard food and water available *ad libitum*. The design of the experiments, housing, handling, care, and processing of the animals were conducted in accordance with the Guide for the Care and Use of Laboratory Animals and the Animal Welfare Act, and all experimental procedures were approved by the Washington University (#22–0254) and Virginia Commonwealth University (#AD10003619) Institutional Animal Care and Use Committees.

### Induction of PHH

**Fig. 1:**
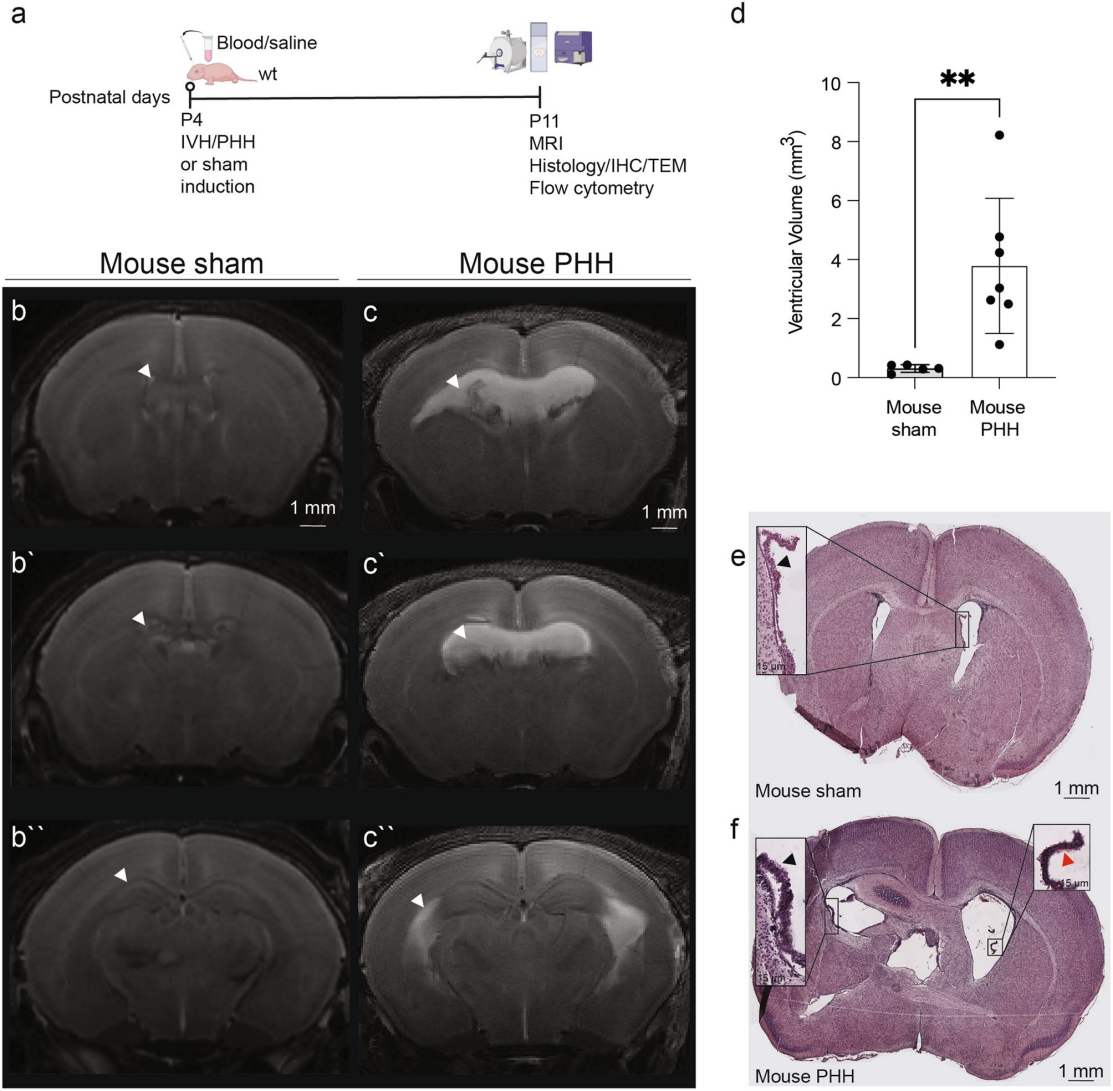
Modeling neonatal PHH through intraventricular injection of lysed blood in mice. **(a)** Sham control and PHH mice received intraventricular injections of saline or lysed blood, respectively, at P4 and analyses were carried out at P11. Representative T2 coronal MRI images of **(b)** sham control and **(c)** PHH mice showing ventriculomegaly after hydrocephalus induction. **(b``-c``)** show serial sections of the same representative cases. Arrows point the lateral ventricles. **(d)** Dot plot showing the volume of the lateral and third ventricles in PHH (*n* = 7) versus sham control (*n* = 5) mice. Means ± SD are shown. **p = 0.0025, two-tailed Wilcoxon–Mann–Whitney test. Hematoxylin and eosin staining of **(e)** sham control and **(f)** PHH representative cases. Magnification shows the ChP in the lateral ventricle (black arrows). The red arrow points to red blood cells observed in the PHH cases. Abbreviations: *IHC*, immunohistochemistry; *MRI*, magnetic resonance imaging; *TEM*, transmission electron microscopy

PHH was induced in neonatal mice at 4 days of age [postnatal day 4 (P4)], equivalent to human infant at gestational week 24 [42]) via bilateral intraventricular injections of 5 µL of lysed blood. To obtain syngeneic blood, littermates were anesthetized, decapitated, and the extracted cardiac blood was then drained into an Eppendorf tube. The tubes were placed in liquid nitrogen for approximately 2 min to lyse the blood cells. As controls, the group of age-matched mice was administered sterile saline (sham-injected control mice). A 26-gauge needle syringe (Hamilton, Cat#75 N, Reno, NV, USA) was used for manual intraventricular injections aimed in the midline between the orbit level and the ear and 1 mm deep to the skull surface following our published methods [43]. A total volume of 5 µL of lysed blood or saline was injected in each lateral ventricle for 30 s. The needle was left for 10 more seconds before removal and mice were allowed to recover from anesthesia on a heated pad and monitored daily until the end of the experiment. PHH cases exhibited lateral ventricular volumes that were above 2 standard deviations from the sham control mean (see Fig. 1d) as previously described in our large animal model [44, 45]. The number of mice per group is indicated in each figure legend.

### Magnetic resonance imaging and lateral ventricle volume analysis

Seven days after lysed blood or saline injections, mice were analyzed by MRI scanning. MRI data were collected using: (1) an Agilent/Varian 4.7-T small-animal MRI scanner equipped with a DirectDriveTM console (Santa Clara, CA, USA), built around an Oxford Instruments (Oxford, UK) horizontal magnet with a 40-cm clear bore diameter; images were collected with a purpose-built actively decoupled transmit (volume, 9-cm inner diameter) and receive (surface, 0.8 cm outer diameter) coil pair; and (2) a 7T (300 MHz) Bruker Biospec MR scanner (Bruker Corporation, MA, USA) with a 30 cm bore diameter; images were collected using a quadrature transmission RF coil of inner diameter 86 mm and an actively decoupled 4-channel parallel array head-only coil (Bruker Corporation, Cat#T11765V3). Mice were anesthetized with Isoflurane/O_2_ (2.5–3.5% isoflurane) in an induction chamber for 5 min. Their heads were placed in prone position and stabilized using a pallet tooth bar inside nosecones for anesthesia, which was maintained at 2–3% during scanning, while temperature was maintained using a heated water recirculating bed and heated air pump.

T1- and T2-weighted transaxial images composed of 21 slices were collected with a 2D fast spin-echo (fsems, VnmrJ 4.2 A) sequence with echo train length (ETL) 4, K_zero_ = 4, TR 2.6 s, effective TE 100 ms, matrix size 128 × 128 and field of view (FOV) 16 × 16 mm^2^ with a slice thickness 0.5 mm, 6 averages and total data collection time 8.5 min. With the Bruker equipment, T2-weighted transaxial images composed with 13 slices were collected using a TurboRARE sequence with rare factor 8, TR 3 s, TE 33 ms; matrix size 256 × 256 and FOV 15 × 15 mm^2^ with slice thickness 0.5 mm, slice gap 0.1 mm, 4 averages, and total data collection time 6 min 24 s in Paravision v7.0.1.

Three-dimensional reconstruction and ventricular volume analyses were performed using sagittal, axial, and coronal MRI images (0.5 mm thick) and the open-source software ITK-SNAP (University of Pennsylvania, US). The lateral and third ventricles were segmented, and the volume was calculated in mm^3^ using the volumes and statistics tools in the software.

### Behavioral tests

All behavioral tests were adapted from [46].

#### Righting reflex (P6 and P8)

The righting reflex is the motor ability of a mouse neonate to be able to flip onto its feet from a supine position. Mice were placed on their backs on a cotton sheet or bench pad and held in position for 5 s. Then, mice were released and the time they took to return to the prone position was recorded. A total of one minute was given for each trial if needed. We repeated a total of three trials.

#### Negative geotaxis (P6, P8)

The negative geotaxis test assesses motor coordination in young mice. Mice were placed facing down a slope and, due to vestibular cues of gravity, neonates turn to face up the slope. Mice were placed with their head pointing downward on a 30-degree incline and held for 5 s. Then, they were released and the time and direction the neonates turned to face upward was recorded. The total testing time was one minute. It was repeated for a total of three trials.

#### Swim tests (P8)

An acryl board was placed on the 2 lids on both sides. Mice were placed in a transparent beaker containing water at a depth of 15 cm maintained at 30–34 °C. We captured the movement of forelimbs and hind limbs with a camera below for 10 s. Two trials were performed. Swimming ability was evaluated by counting the alternating frequency of the hind limbs.

### Flow cytometry

Working under a stereomicroscope, brains were dissected and deposited in ice-cooled Petri dish with Roswell Park Memorial Institute (RPMI) medium 1640 (ThermoFisher Scientific, Cat#11875085, Waltham, MA, USA). ChP were dissected and digested at 37 °C for 30 min with collagenase D (400 units/ml; Sigma-Aldrich - Roche, Cat#11088858001, St Louis, MO, USA), collagenase VIII (400 units/ml, Sigma-Aldrich, Cat#C2139), and 50 µg/ml of DNase I (Sigma-Aldrich, Cat#DN25) in RPMI medium. ChP were then mechanically dissociated with a glass Pasteur pipette, filtered through a 70-µm nylon cell strainer (BD Bioscience, Cat# 340635, San Jose, CA, US), and centrifuged at 300 g for 10 min. Cells were resuspended in 100 µl of cytometry buffer containing 0.5% bovine serum albumin (BSA, Sigma-Aldrich, Cat# A7030), 5 mM EDTA (ThermoFisher Scientific-Invitrogen, Cat#AM9260G, Burlington, MA, USA) in PBS (0.1 M, pH 7.4). Cells were incubated for 10 min at room temperature with Zombie NIR Fixable Viability Kit (1:2000, BioLegend, Cat#423105, San Diego, CA, USA) to assess viability. Then, cells were washed with cytometry buffer and blocked with FcR blocking reagent (1:50, Miltenyi Biotec, Bergisch Gladbach, Germany). Samples were then washed with cytometry buffer and incubated for 1 h at room temperature with the primary antibodies (Suppl Table 2). This panel was titrated and optimized with all surface markers using 0.5 µl of each antibody and fluorescence minus one (FMO) control. All samples were stained in the presence of Brilliant Stain Buffer (BD Bioscience, Cat# 563794), per manufacturer protocol. Cells were then centrifuged at 400 g for 5 min, washed with PBS (0.1 M, pH 7.4), fixed for 7 min, and permeabilizated overnight with Fixation/Permeabilization solutions following manufacturer instructions (ThermoFisher Scientific - Invitrogen, Cat#005223-56, Cat#00-5123-43, and Cat#00-8333-56).

Samples were analyzed on a Cytek Aurora Spectral CSF flow cytometer (Cytek Bioscience, Fremont, CA, US) using Cytek Spectroflow Software (Cytek Bioscience). Analyses were performed using FlowJo Software v10.8.2 (FlowJo LLC, Ashland, OR US). Live cells were identified by their negative staining for the live/dead cell marker (Zombie NIR) in comparison to FSC-A. Single cells were gated based on their FSC-A and FSC-H parameters. Macrophages were identified by the following cell markers: CD45^+^, CD11b^+^, F4/80^+^, and CD206^+^. Populations were gated based on fluorescence minus one and unstained controls (Suppl. Figure 1).

### Fluorescence-activated cell sorting

Spleens were dissected from adult mice (~ 7 weeks) and cells were collected following our previous published methods [47]. Briefly, spleens were mechanically dissociated, and cells were filtered through a 70 μm cell strainer and centrifuged at 500 g for 10 min. Cell suspensions were incubated with red blood lysis buffer (ThermoFisher Scientific, Cat#J62990.AP) for 5 min at 4 °C. Then, cells were incubated with anti-CD11b, anti-Ly6C, and anti-Ly6G antibodies (Suppl. Table 2). CD11b^+^, Lys6C^+^, and Ly6G^−^ cells were sorted using BD FACSAria Fusion SORP sorter (BD Bioscience) placed in BSL2 biosafety cabinet and collected into 20% fetal bovine serum (FBS, ThermoFisher Scientific, Cat# A5256801) in Dulbecco’s Modified Eagle Medium (DMEM, ThermoFisher Scientific, Cat#11965092). Sorted cells were validated with 99.1% purity.

### In vitro ChP cultures

After euthanasia, brains were dissected, and ChP explants were collected from P4 mice. The explants were placed in 24-well plates containing 0.5 ml of culture medium consisting of DMEM/F12 (Gibco, Cat#113–200), 10% FBS, and 0.2 µg/ml of Cytosine β-D-arabinofuranoside hydrochloride (Sigma-Aldrich, Cat#C6645). Twenty-four hours later, the following treatments were administered: (a) 25 µl of lysed blood (*n* = 5), (b) 25 µl of saline (*n* = 4), (c) 25 µl of lysed blood combined with 10,000 CD11b^+^ Ly6G^−^ Ly6C^+^ cells (*n* = 7), or (d) 25 µl of saline combined with 10,000 CD11b^+^ Ly6G^−^ Ly6C^+^ cells (*n* = 6). The number of CD11b^+^ Ly6G^−^ Ly6C^+^ cells was based on our previous studies using similar co-culture systems of ventricular zone cells and astrocytes [48]. Blood or saline and CD11b^+^ Ly6G^−^ Ly6C^+^ cells were added to 0.5 ml of medium containing one ChP explant per well. The samples were incubated for an additional 24 h before analyses.

### Histology and immunofluorescence

Mice were anesthetized with Isoflurane/O_2_ (3.5% isoflurane) in an induction chamber and euthanized. Brains were immersion-fixed in 4% paraformaldehyde (Sigma-Aldrich, Cat#158127) in PBS (0.1 M, pH 7.4) for 48 h at room temperature. Fixed brains were dehydrated, embedded in paraffin (Fisher Scientific, Cat#22900700), and sectioned serially in a microtome (Leica HistoCore biocut Manual Rotary Microtome, Wetzlar, Germany) at a thickness of 10 μm. Heat-induced antigen retrieval in citrate (50 mM, pH 6.0) was performed for 10 min. ChP explants were fixed in 4% paraformaldehyde for 10 min at room temperature.

Brain or ChP samples were placed in PBS (0.1 M, pH 7.4) containing 0.1% Triton X-100 (Sigma-Aldrich, Cat#X100) and BSA 1% for 45 min at room temperature on a rocker. Afterwards, primary antibodies (Suppl. Table 2) were incubated for 18 h at 22 °C or 72 h at 4 °C. Secondary antibodies were conjugated with Alexa Fluor 488, Alexa Fluor 555, or Alexa Fluor 647 (1:500, ThermoFisher Scientific) for 1 h at room temperature. 2-(4-aminophenyl)-1 H-indole-6-carboxamidine (DAPI, ThermoFisher Scientific - Molecular Probes Life Technology, Cat#D1306) was used for nuclear staining at a 1:5000 dilution in PBS (0.1 M, pH 7.4) for 5 min. Due to host-species constraints when co-immunostaining with rabbit anti-Iba1, human post-mortem tissues were labeled with mouse anti-Claudin-5 (Invitrogen, Cat#35-2500; dilution 1:50) to avoid secondary antibody cross-reactivity. Primary antibodies were diluted in PBS (0.1 M, pH 7.4) containing 0.1% Triton X-100 and 1% BSA. Slides were mounted with a water-based mountain medium (Fluoromount-G Mounting Medium, ThermoFisher Scientific, Cat#00-4958-02).

For histology, paraffin sections were deparaffinized and hydrated. Hematoxylin (Harris, Cat#061920A2, Biocare Medical, CA, USA) was applied for 7 min and sections were rinsed for 5 min in running tap water and then differentiated via a quick dip in 0.5% acid ethanol (90% ethanol, 0.5% acetic acid). 0.5% eosin (Sigma-Aldrich, Cat#1.09844.1000) was applied for 3 min and then sections were dehydrated and mounted in xylene-based medium (Epredia Cytoseal XYL Mountant, Fisher Scientific, Cat#8312-4).

### Transmission electron microscopy

TEM brain samples were immersion-fixed overnight at 4 °C in a solution containing 2% paraformaldehyde and 2.5% glutaraldehyde (Sigma-Aldrich) in 0.15 M cacodylate buffer (Sigma-Aldrich) with 2 mM calcium chloride (Sigma-Aldrich), pH 7.4. Samples were then rinsed in cacodylate buffer 3 times for 10 min each and subjected to a secondary fixation for 1 h in 2% osmium tetroxide (Sigma-Aldrich) with 1.5% potassium ferrocyanide (Sigma-Aldrich) in cacodylate buffer. Following this, samples were rinsed in ultrapure water 3 times for 10 min each and stained overnight in an aqueous solution of 1% uranyl acetate at 4˚C. Samples were then washed in ultrapure water 3 times for 10 min each, dehydrated in a graded acetone (Sigma-Aldrich) series (50%, 70%, 90%, 100% x4) for 15 min in each step, and infiltrated with microwave assistance (Pelco BioWave Pro, Redding, CA, USA) into Spurr’s resin (Electron Microscopy Sciences, Cat#14300, Hatfield, PA, USA). Samples were then cured in an oven at 60 °C for 80 h. 70 nm thin sections were next cut from the resin block, post-stained with uranyl acetate and Sato’s lead, and imaged on a Transmission Electron Microscope (JEOL JEM-1400 Plus, Tokyo, Japan) operating at 120 KeV.

### Image analysis and quantification

Immunofluorescence images (1024 × 1024-pixel resolution) of the ChP were obtained with a Zeiss LSM 880 Airyscan Two-Photon Confocal Microscope, Zeiss AxioImager Z2 Fluorescence Microscope with ApoTome 2 (Oberkochen, Germany), and Leica CTR5500 fluorescent microscope. Bright-field micrographs were obtained with a Zeiss Axio Scan Z1 Brightfield Slide Scanner (Oberkochen, Germany). Representative images for the figures were obtained under the confocal microscope, and Z-stack of 10 μm (1 μm-thickness) for brain tissue sections and 14 μm (1 μm-thickness) for ChP explants were composed with Fiji Software, version 2.14.0/1.54f (ImageJ, Madison, WI, USA). For TEM, we systematically analyzed the choroid plexus from 3-5 animals per group, and processed at least 4 grids per case. For each experiment, images were obtained in batches using the same settings. Figures were composed using Adobe Illustrator (Mountain View, CA, USA). Up to six non-overlapping immunofluorescence images covering the entire ChP in each lateral ventricle within one randomly-selected 10 μm-thick section were taken for each animal and quantified. The number of macrophages (Iba1^+^ cells and CD68^+^ cells) in the ChP in the lateral ventricle were quantified as a fraction of the total ChP cell nuclei (DAPI) using Fiji. The number of neurons (NeuN^+^ cells) and cells in apoptosis (cleaved caspase 3^+^ cells, with and without NeuN) in the neocortical layers II-III and VI were quantified as a fraction of the total cell nuclei (DAPI) using Fiji Software (See Suppl. Figure 1e). Total DAPI^+^ cells in neocortical layers II-III and VI were quantified by area (10,000 µm^2^). ZO-1 and claudin-1 membrane positive signals in ChP epithelial cells were quantified based on staining intensities according to set thresholds using Fiji Software. To isolate junctional signaling from non-specific cytoplasmic fluorescence, we employed a spatial masking protocol in Fiji: a binary mask was generated by applying a thresholding algorithm to the Claudin-1/ZO-1 channels, eliminating the background. Groups were blinded for all analyses.

### Statistical analysis

Statistical analyses were performed using GraphPad Software (San Diego, CA, USA). Samples were numbered without indication of the group. All values are reported in the figures as mean ± standard deviation (SD). The Wilcoxon–Mann–Whitney test was applied for hypothesis testing in non-parametric analyses. Normality was previously analyzed by normality tests (Anderson-Darling test, D’Agostino-Pearson omnibus normality test, Shapiro-Wilk normality test, Kolmogorov-Smirnov normality test with Dallal-Wilkinson-Lillie for *P* value). Simple linear regressions were used to study correlations between ventricular volume, Iba1^+^ and CD68^+^ cells, and fluorescent intensity of ZO-1 and claudin-1 in the ChP of PHH cases. *P* < 0.05 based on both tests was considered statistically significant.

## Results

### Modeling neonatal PHH through intraventricular injection of lysed blood

To elucidate cell junction pathology and associated immune reaction in PHH ChP, we used a mouse model of PHH by administrating lysed blood [49, 50] in both lateral ventricles (Fig. 1a).

Ventriculomegaly elicited by injection of lysed blood was confirmed by MRI and histology (Fig. 1b-f). Animals that underwent 5 µl of lysed blood injections developed ventriculomegaly when assessed via MRI (Fig. 1b-c``), with significantly larger (3.8 ± 2.3 mm^3^; *p* = 0.0025) ventricles at 7 days post-induction compared to sham control animals (0.3 ± 0.1 mm^3^; Fig. 1d). PHH was defined as ventricular size 2 standard deviations above the sham control mean (Fig. 1d), as previously described in our large animal model [44, 45]. Hematoxylin and eosin-stained sections also showed ventricular expansion after the injection of 5 µl of lysed blood compared to saline (sham) controls (Fig. 1e, f).

Mortality associated with the procedure was not observed. Mice could survive at least 30 days post-induction, still displaying ventriculomegaly (Suppl. Figure 1a). PHH induction did not cause significant differences in body weight and no deficits were observed when we performed a battery of behavioral tests for neonates (adapted from [46], see Methods; Suppl. Figure 1b-d).

To analyze if the procedure to induce PHH affects brain tissue viability, we performed DAPI staining across neocortical layers II-III (far from the lateral ventricle) and VI (close to the lateral ventricle) to screen for significant changes in nuclear density (Suppl. Figure 1e). Our analysis revealed no significant differences in the total number of DAPI^+^ cells per µm^2^ across the neocortical layers (Suppl. Figure 1f-h). Similarly, the percentage of NeuN^+^ neurons in those neocortical regions were similar between groups (Suppl. Figure 1 m-o). To detect more subtle markers of distress, we further quantified neocortical cellular health using cleaved caspase 3 and NeuN (Suppl. Figure 1p-s) finding no differences between groups (Suppl. Figure 1t, u). The stability of NeuN^+^ populations and the absence of significant caspase 3 activation—coupled with intact DAPI cytoarchitecture—collectively suggest that the procedure did not compromise brain tissue viability.

### Macrophages are enriched in the ChP in PHH

Since the capillaries in the ChP stroma are fenestrated, and therefore do not have tight junctions [51, 52], the integrity of the ChP epithelium is key to maintain the blood-ChP-CSF barrier function to prevent immune cell trafficking and other paracellular diffusion to the CSF [43, 51]. Macrophages have been implicated in tight junction integrity and disruption of epithelial barriers [53, 54]. We therefore investigated the role of ChP macrophages in our mouse model and human post-mortem samples.

**Fig. 2:**
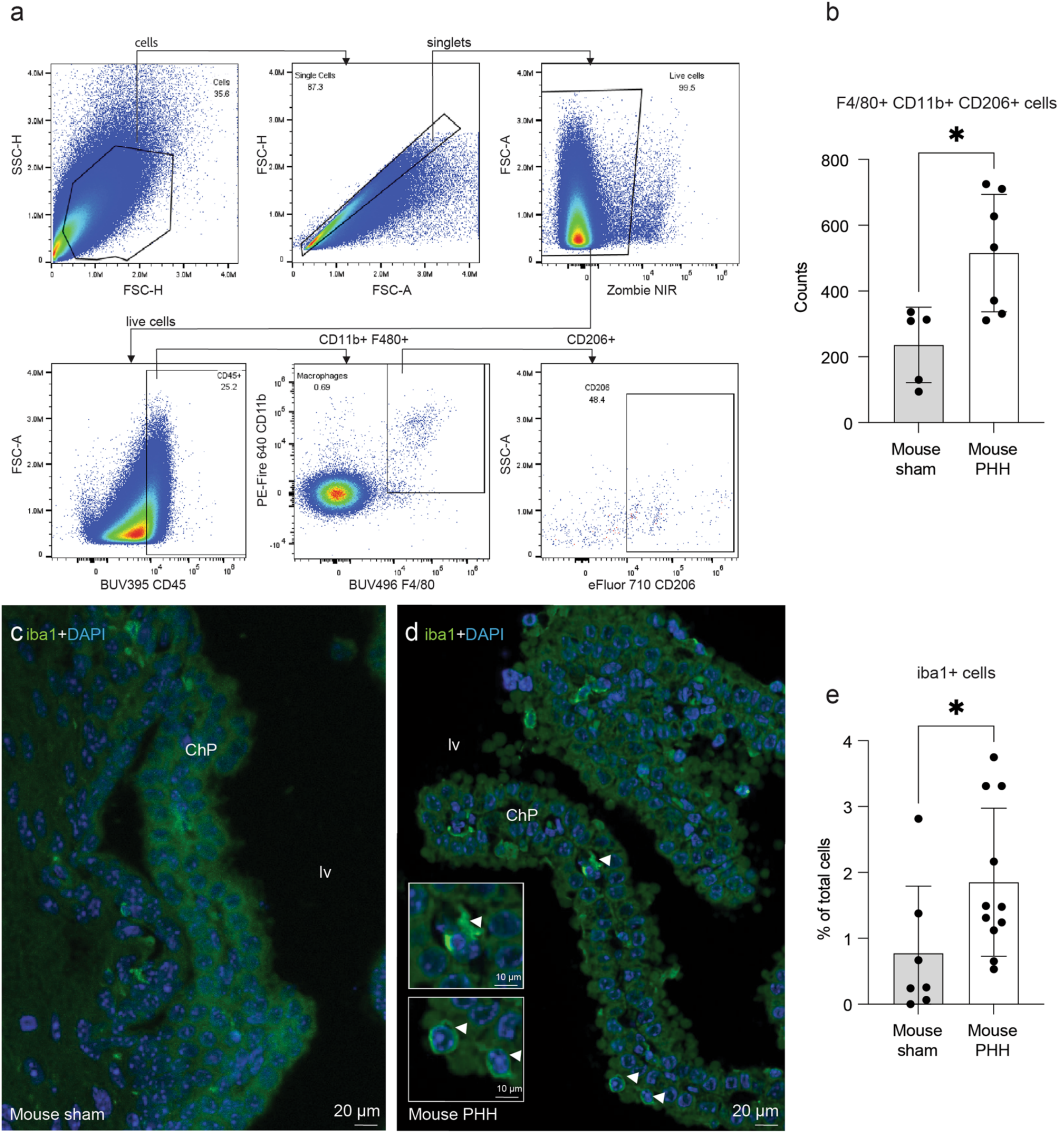
The number of macrophages is elevated in the ChP of mice with PHH. (a) Gating strategy to identify F4/80^+^, CD11b^+^, CD206^+^ macrophages in ChP samples. (b) Dot plot showing an increase in macrophages in PHH mice. Means ± SD are shown. *p=0.0177, two-tailed Wilcoxon -Mann -Whitney test. For the flow cytometry experiments, n=5 and n=7 cases were used for the sham control and PHH groups, respectively. Immunofluorescence of the ChP using Iba1 (fluorescence in green) in (c) a sham control and (d) a PHH mouse. An arrow pointing towards the area of ChP magnification represents an Iba1^+^ cell in the ChP in PHH. (e) Dot plot for iba1 quantification in the ChP showing an increased in macrophages in PHH (n=11) compared to sham control (n=7). The number of Iba1^+^ cells in the ChP in the lateral ventricle was quantified as a percent of the total number of ChP cells, identified by DAPI^+^ nuclei. Means ± SD are shown. *p=0.0346, two-tailed Wilcoxon -Mann -Whitney test. DAPI stained all nuclei in blue. Abbreviations: ChP choroid plexus; lv, lateral ventricle

Using standard markers and spectral flow cytometry, ChP macrophages were identified as CD45^+^, F4/80^+^, CD11b^+^, and CD206^+^ [3, 55, 56] (Fig. 2a, Suppl. Figure 2), in contrast to microglia, which are located in other brain compartments and do not have CD206 expression [37, 57].

**Fig. 3:**
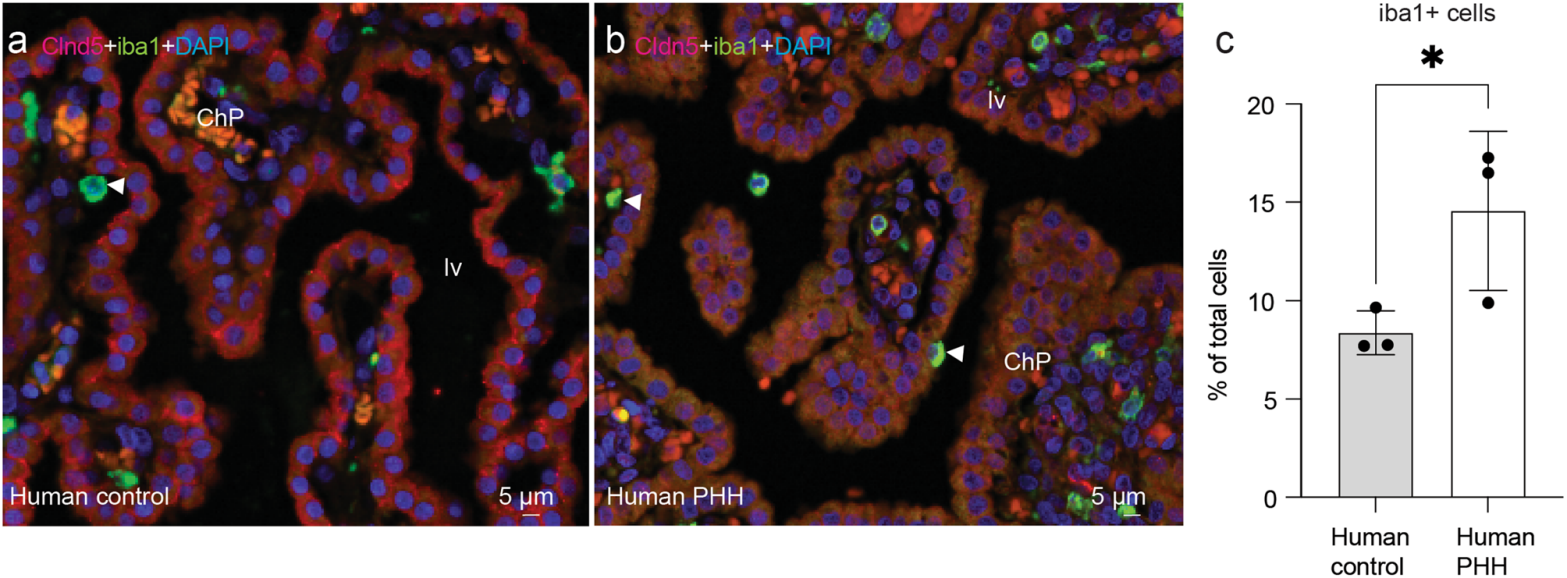
Iba1^+^ macrophages in human post-mortem ChP. The number of macrophages was increased in the human ChP in (**b**) PHH compared to (**a**) control when assessed using claudin-5 (fluorescence in *red*) and Iba1 (fluorescence in *green*, white arrows). **(c)** Dot plot of Iba1^+^ quantification in *n* = 3 samples per group. The number of Iba1^+^ cells in the ChP in the lateral ventricle were quantified as a percent of the total number of ChP cells, identified by DAPI^+^ nuclei. Means ± SD are shown. *p≤0.05, one-tailed Wilcoxon–Mann–Whitney test. DAPI stained all nuclei in blue. Abbreviations: *ChP*, choroid plexus, *lv*, lateral ventricle

Mouse PHH ChP displayed a significant enrichment (*p* = 0.0177) of CD45^+^, CD11b^+^, F4/80^+^, CD206^+^ macrophages (515 ± 178 cells) compared to controls (236 ± 115 cells, Fig. 2b). The increase in the overall percentage of macrophages in mice with PHH compared to controls was confirmed by immunofluorescence using Iba1^+^ [5, 37] (1 ± 1% in sham controls vs. 2 ± 1% in PHH, *p* = 0.0346; Fig. 2c-e). Notably, macrophages were located within the stroma and at the apical surface of the ChP epithelial cells (Fig. 2d). This increase in macrophages in PHH ChP was also corroborated in the human post-mortem samples (Fig. 3a, b). Immunohistochemical analysis revealed that the percentage of Iba1^+^ cells was significantly increased (*p*≤0.05) in human PHH (15 ± 4%) compared to controls (8 ± 1%; Fig. 3c). Similar to mice, macrophages were also located within the stroma ant the surface of the ChP epithelial cells (Fig. 3b).

### Macrophages display active endocytic activity in PHH ChP

**Fig. 4:**
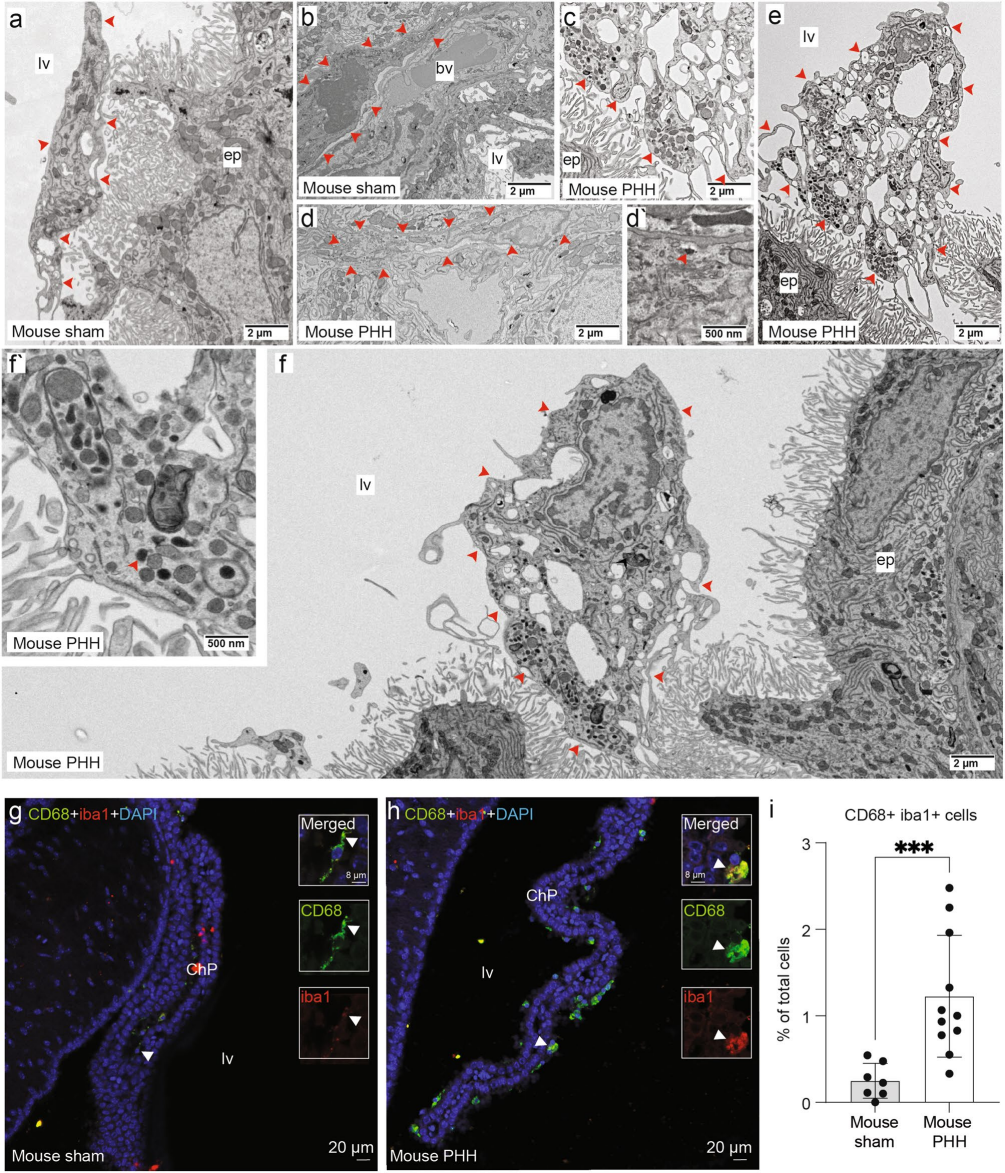
Macrophages display active endocytic activity in the ChP of mice with PHH. TEM image from a sham control showing **(a)** a macrophage (phagocytic cell containing lysosomes, phagosomes, and residual bodies) adjacent to an epithelial cell and **(b)** within the ChP stroma (*arrows* showing the boundaries of both cells in a and b). Representative TEM images from PHH cases showing **(c)** activated macrophages at the surface of the ChP epithelium and **(d)** within the ChP stroma (*arrows* showing the boundaries of the cells in c and d). **(d`)** Higher magnification of the cytoplasm of the stromal macrophage from a PHH mouse showed in **d**. Arrow points a possible lysosome. **(e)** Macrophages (*arrows* showing the boundaries of the cells) displayed an irregular shape with multiple cell protrusions suggesting their activation in PHH. **(f)** Lysosomes and phagosomes in the cytoplasm of the activated PHH macrophage were observed (arrows showing the boundaries of the cells) and are shown in more detail in the higher magnification image in **(f`)**. *N* = 3 sham controls and *N* = 5 PHH samples were observed by TEM. Representative micrographs of a control (**g**) and a PHH (**h**) mouse immunostained with iba1 and CD68. Details of the double positive cells pointed with white arrows are shown on the right. (**i**) Bar plot of the iba1^+^ CD68^+^ cell quantification. The % of Iba1^+^ CD68^+^ cells in the ChP in the lateral ventricle was quantified from the total number of ChP cells, identified by DAPI^+^ nuclei. *N* = 7 sham controls and *n* = 11 PHH mice were used. Means ± SD are shown. ***p = 0.0003, two-tailed Wilcoxon–Mann–Whitney test. Abbreviations: *bv*, blood vessel; *ep*, ChP epithelium, *lv*, lateral ventricle

The main function of macrophages is to ingest and degrade a variety of particles via endocytic, phagosomal, and lysosomal activities [36, 58]. After stimulation and activation, macrophages become irregularly shaped, displaying a large number of membrane extensions, and containing an increased number of phagosomes and lysosomes in their cytoplasm [59]. Therefore, we next assessed whether these ChP macrophages were activated in IVH/PHH by analyzing their ultrastructure by TEM (Fig. 4a-f).

We observed macrophages at the apical surface of the ChP epithelial cells on the ventricular side and within the stroma (Fig. 4a, b control, and 4c-f PHH), in accordance with the observations made by immunofluorescent staining. In the control group, a mean of 1.7 ± 1.5 activated macrophages were observed by TEM (a total of 5), while in the PHH group, 6.2 ± 9 activated macrophages were counted by TEM (a total of 31). Further, in PHH, the macrophages displayed a more irregular shape with multiple cell protrusions, and multiple lysosomes and phagosomes in the cytoplasm (see inserts in Fig. 4d and f), suggesting a more activated state.

To validate the results observed by TEM, we used CD68, a marker for endosomes/lysosomes highly expressed in macrophages [60, 61]. Immunohistochemical methods revealed a significant increase (*p* = 0.0003) in the percentage of iba1^+^ CD68^+^ cells in the ChP of PHH mice (1.2 ± 0.7%) compared to sham (0.2 ± 0.2%; Fig. 4g-i).

### Decrease in tight junctional proteins after PHH in the ChP epithelium

The blood-ChP-CSF barrier, formed by ChP epithelial cells, provides a robust protective barrier for the brain [62]. It is well established that the tight junctions between ChP epithelial cells are the primary intercellular junctions that maintain the integrity of the blood-ChP-CSF barrier [43]. Due to the increased number of activated macrophages in PHH ChP, we analyzed the tight junction proteins ZO-1 and claudin-1 to evaluate the integrity of the ChP epithelium barrier.

**Fig. 5:**
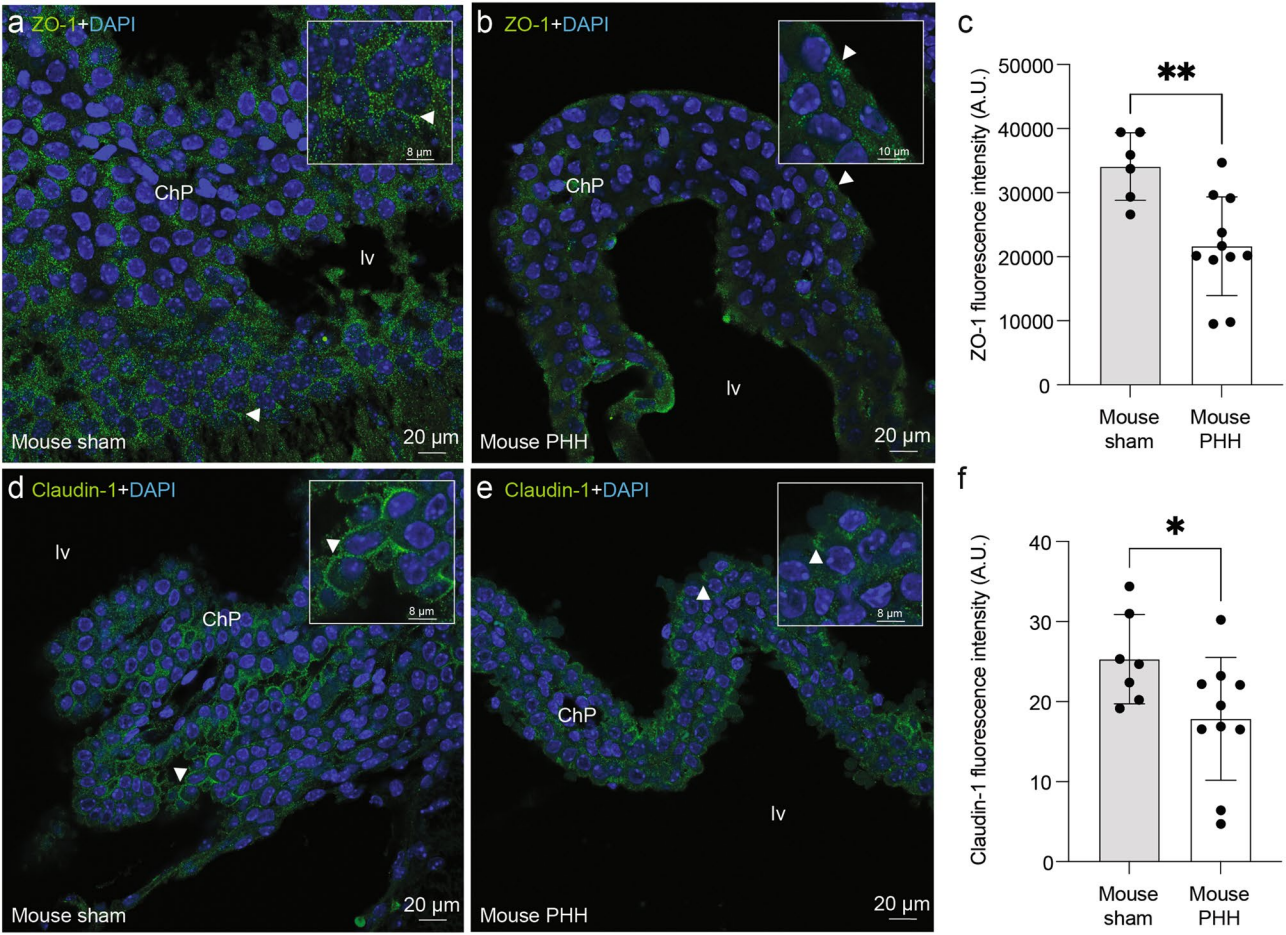
Decrease in junctional proteins in the ChP epithelium in the mouse model of PHH. Immunofluorescent images of the ChP tight junction protein ZO-1 (*green*) in **(a)** sham control and **(b)** PHH representative cases. **(c)** Dot plot showing a significant decrease in ZO-1 fluorescent intensity in PHH (*n* = 10) compared to sham control (*n* = 6). Means ± SD are shown. ** *p* = 0.0048, two-tailed Wilcoxon–Mann–Whitney test. Claudin-1 immunofluorescent images (*green*) in **(d)** sham control and **(e)** PHH representative cases. **(f)** Dot plot showing the fluorescent intensity of claudin-1 in PHH (*n* = 7) compared to sham control (*n* = 10). Means ± SD are shown. *p = 0.033, two-tailed Wilcoxon–Mann–Whitney test. In **a**, **b**, **d**, **e**, arrows point towards the area of ChP magnification. 1-µm thick sections are shown. DAPI stained all nuclei in blue. Abbreviations: *ChP* choroid plexus; *lv*, lateral ventricle.

We observed delocalization and significant reduction in the tight junction proteins ZO-1 (*p* = 0.0048; Fig. 5a-c) and claudin-1 (*p* = 0.033; Fig. 5d-f) in the ChP epithelium in mice with PHH (21642 ± 7715 and 17.8 ± 7.7, respectively) compared to sham control mice (34082 ± 5273 and 25.3 ± 5.6, respectively).

**Fig. 6:**
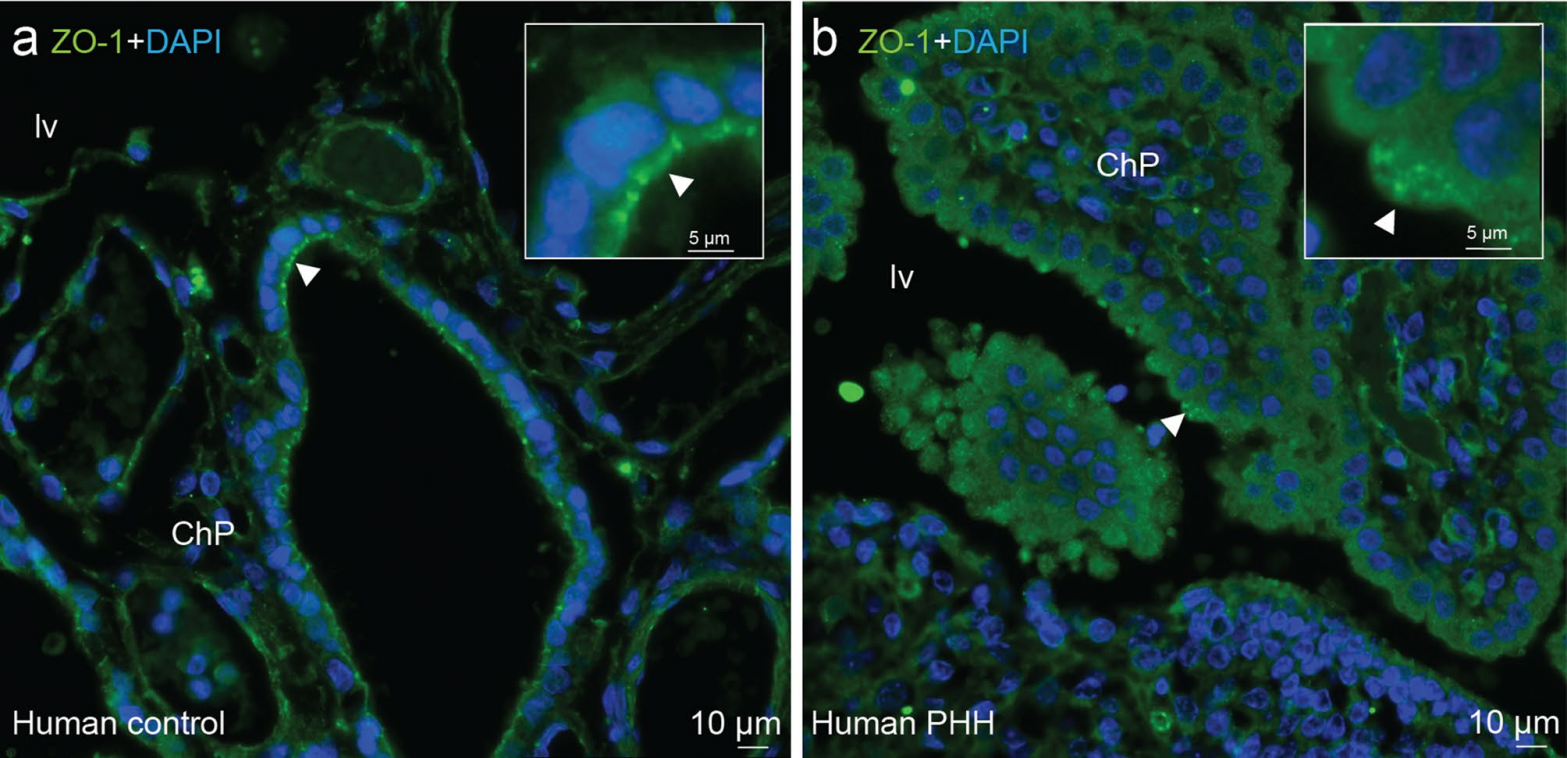
Decrease in junctional proteins in the ChP epithelium in human post-mortem cases. Immunofluorescence of the ChP tight junction protein ZO-1 (fluorescence in *green*) in **(a)** control and **(b)** PHH representative human post-mortem cases. Arrows point towards the area of ChP magnification. Note the mislocation of ZO-1 in the PHH case where the tight junction protein is not well located along the cell surface as in the control, but still present in the cytoplasm. *n* = 2 cases per group were observed. DAPI stained all nuclei in blue. Abbreviations: *ChP* choroid plexus; *lv*, lateral ventricle.

ZO-1 findings were corroborated by observations in post-mortem human ChP samples, where it was found to be delocalized in the ChP epithelium of PHH subjects (Fig. 6), while claudin-1 staining was equivocal. ZO-1 immunoreactivity in human control cases was in the cell surface (Fig. 6a), while in PHH subjects ZO-1 was in the cytoplasm (Fig. 6b).

**Fig. 7:**
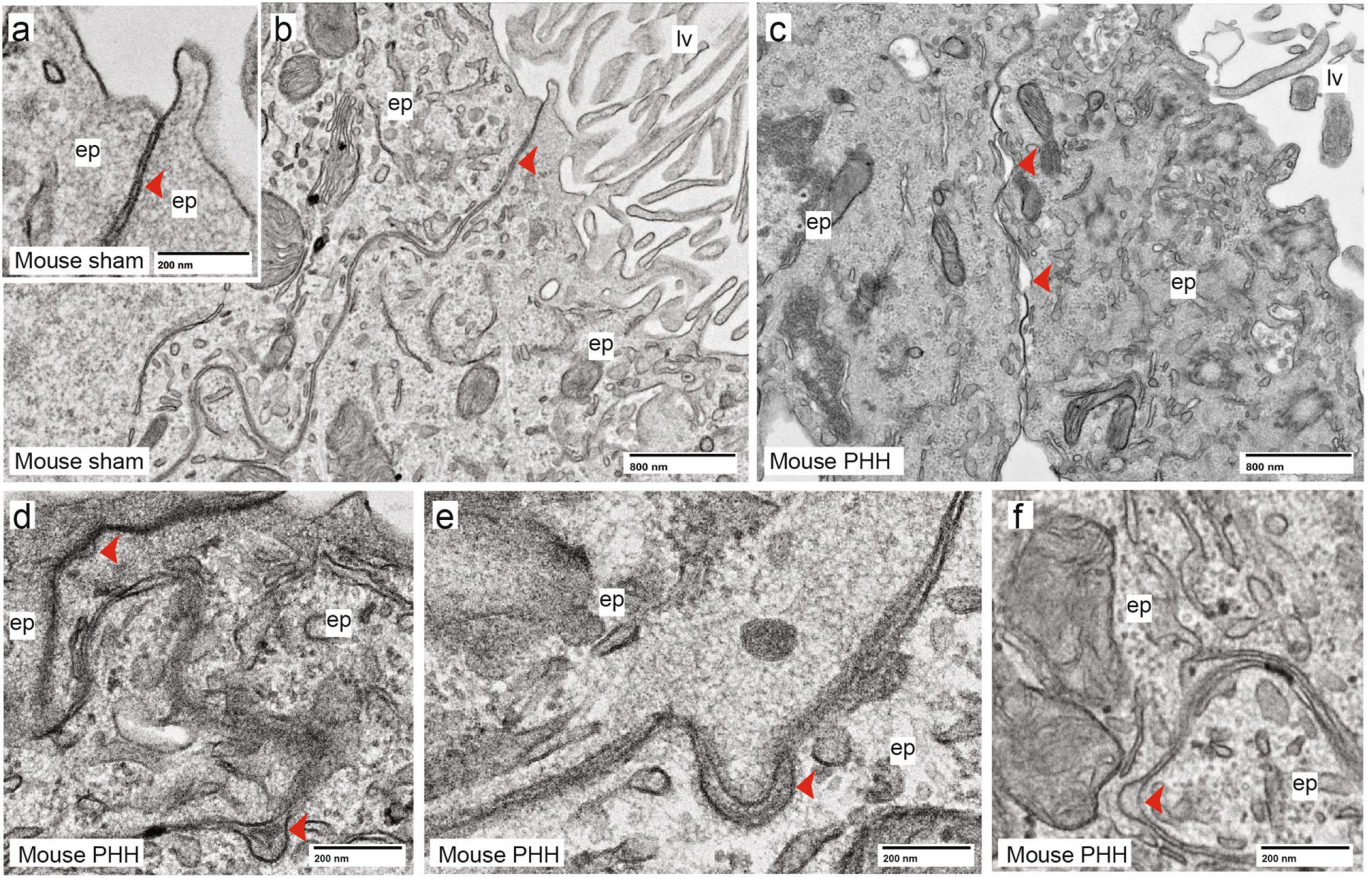
Ultrastructure of the ChP epithelial cells in sham control and PHH mice. TEM images showing tight junctions between epithelial cells (*ep*) in **(a)** a sham control, detail with higher magnification in **(b)**, indicated by the *arrow*. **(c)** PHH cases show gaps between cells (*arrows*). **(d)** shows a detail of a tight junction in PHH (top *arrow*) and the gaps observed between cells (bottom *arrow*). Notably, gaps between cells were observed in PHH suggesting impairment of cell-cell attachment, as further displayed in **(e)** and **(f)**. *n* = 5 cases per group were used. Abbreviations: *ep*, epithelial cell; *lv*, lateral ventricle.

We next investigated whether this ZO-1 and claudin-1 impairment was affecting the ultrastructure of the ChP tight junctions by TEM. Using the same sample numbers and grids described above, we observed sham controls (Fig. 7a, b) and PHH samples (Fig. 7c, d). In PHH samples, TEM revealed gaps were identified between cells (Fig. 7e, f) suggesting am impairment of cell-cell attachment.

These changes in tight junction proteins and Iba1^+^ and CD68^+^ cells seem to not be associated with ventricular volume (Suppl. Figure 3a-d), and no significant correlations were found between those markers (Suppl. Figure 3e-h).

### Infiltrating CD11b^+^ Ly6G^−^ Ly6C^+^ cells are correlated with ChP ZO-1 disruption

As our data indicated that an increased number of macrophages and tight junction disruption may be linked, we develop an in vitro model of ChP to test if the macrophages are involved in ChP tight junction breakdown in PHH (Suppl. Figure 4a). Since one of the subpopulations of macrophages are generated from blood-derived CD11b^+^ Ly6G^−^ Ly6C^+^ cells (monocytes) [36, 63], and the spleen is a reservoir of peripheral monocytes that allows the isolation of high numbers for in vitro studies [64], we isolated peripheral CD11b^+^ Ly6G^−^ Ly6C^+^ cells from the spleen and co-cultured 10,000 cells with one mouse ChP explant per well (Suppl. Figures 5 and 6).

**Fig. 8:**
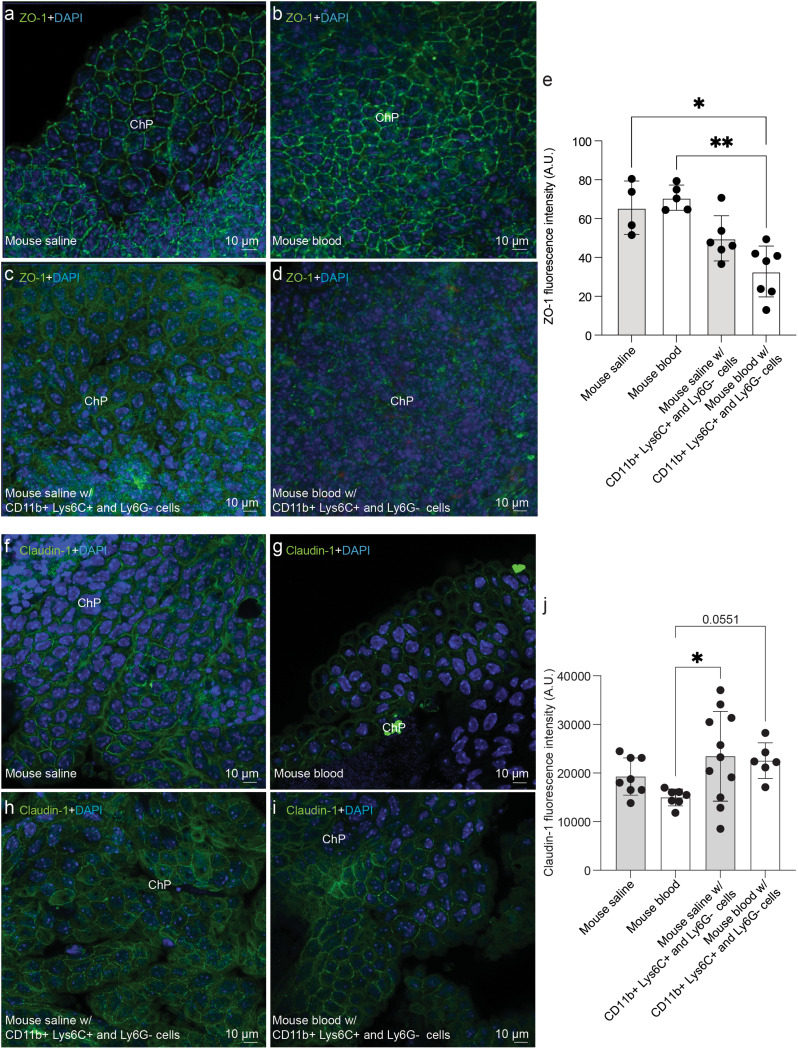
CD11b^+^ Ly6G^−^ Ly6C^+^ cells co-cultured with mouse ChP in vitro. ZO-1 and claudin-1 immunofluorescent images of in vitro ChP cultures exposed to **(a**,** f)** saline, **(b**,** g)** lysed blood, **(c**,** h)** CD11b^+^ Ly6G^−^ Ly6C^+^ cells and saline, and **(d**,** i)** CD11b^+^ Ly6G^−^ Ly6C^+^ cells and lysed blood. **(e)** Dot plot showing significant differences in ZO-1 fluorescent intensity between ChP exposed to lysed blood with CD11b^+^ Ly6G^−^ Ly6C^+^ cells (*n* = 7), saline (*n* = 4), and lysed blood (*n* = 5). No differences were found between ChP exposed to lysed blood with CD11b^+^ Ly6G^−^ Ly6C^+^ cells and saline with CD11b^+^ Ly6G^−^ Ly6C^+^ cells (*n* = 6). **(j)** Dot plot showing significant differences in claudin-1 intensity between ChP exposed to saline (*n* = 8), lysed blood (*n* = 7), saline with CD11b^+^ Ly6G^−^ Ly6C^+^ cells (*n* = 11), and lysed blood with CD11b^+^ Ly6G^−^ Ly6C^+^ cells (*n* = 6). Means ± SD are shown. **p = 0.0046, *p = 0.0233 for ZO-1, and *p = 0.0391 for claudin-1. Kruskal-Wallis test corrected for multiple comparison with Dunn’s test. DAPI stained all nuclei in blue. Abbreviations: *ChP*, choroid plexus.

We confirmed that ChP maintained their structure, tight junctions (claudin-1, ZO-1), cilia (βIV tubulin), and water transit [aquaporin-1 (AQP1); Suppl. Figure 4b, c] when cultured in vitro for 48 h. Then, we exposed them to lysed blood or saline, and did not observe significant differences in ZO-1 intensity (Fig. 8a, b). However, when ChP were co-cultured with CD11b^+^ Ly6G^−^ Ly6C^+^ cells and lysed blood, tight junction ZO-1 levels (32.79 ± 13.11) were significantly reduced (*p* = 0.0046) compared to ChP co-cultured with CD11b^+^ Ly6G^−^ Ly6C^+^ cells and saline (49.8 ± 11.64; Fig. 8c-e). By contrast, claudin-1 intensity was reduced when ChP were cultured only with blood (14979 ± 1718 blood, 23446 ± 9223 saline + CD11b^+^ Ly6G^−^ Ly6C^+^ cells, *p* = 0.0391), but did not show significant changes when ChP were co-cultured with CD11b^+^ Ly6G^−^ Ly6C^+^ cells and lysed blood (Fig. 8f-j).

These data suggest that there is a correlation between CD11b^+^ Ly6G^−^ Ly6C^+^ cells and ChP ZO-1 tight junction disruption.

## Discussion

This study provides a detailed investigation of mouse and human neonatal ChP junctional biology and immune reaction. The results implicate tight junction disruption of the ChP epithelial cells together with activated macrophages in the pathophysiology of PHH. Our data suggest macrophages as mediator of this inflammatory response and ChP tight junction impairment. These data corroborate and significantly extend observations previously made in other models of hydrocephalus [4, 6, 65, 66] and suggest PHH is a neuroimmune disorder, similar to other infectious and neurodegenerative conditions [67–70].

Existing animals models for PHH have been developed using different compounds, including collagenase to cause direct injury to the germinal matrix [71, 72], glycerol to affect intracranial hypotension [73], or iron to analyze the role of this specific blood component [74]. Our mouse model of PHH through bilateral intraventricular injections of lysed blood, similar to other models in rats [75, 76], resembles the human condition and allowed us to study the inflammatory impact of blood components within the ventricles. IVH is diagnosed in up to 38% of preterm infants born at less than 28 weeks of gestational age, and for those with severe IVH, the risk of developing PHH is 40–50% [77]. Our mouse model for PHH induction at P4 corresponds to a human gestational age of approximately 24 weeks [42], allowing for a proper alignment with human neurodevelopment and aging. Unilateral injection can lead to asymmetric ventriculomegaly and precaution should be taken when considered evidence for treatment of hydrocephalus as it may reflect tissue damage, rather than impaired CSF dynamics [78]. By utilizing bilateral injections, we reduce the risk that the observed ventriculomegaly reflects focal tissue damage, rather than a ventricular system-wide process. Clinically, blood and blood-breakdown products are often located in both ventricles [79]. Bilateral injections may thus reduce the reliance on variable CSF distribution, essential for models implementing unilateral injections. Also, our data suggest that bilateral injections are not causing neocortical tissue damage since total DAPI^+^ cells, NeuN^+^ neurons, and cleaved caspase 3^+^ cells were similar between groups.

The blood-ChP-CSF barrier is formed by the epithelial cells joined by tight junctions in their apical membrane and is crucial for the homeostatic regulation of the brain environment [80]. They permit the establishment of unique environments in opposing compartments [81]. Our results showed a decrease in the levels of tight junction proteins ZO-1 and claudin-1, known markers of tight junctions at the blood-ChP-CSF barrier [82], after PHH induction. Although there are other claudins in the ChP epithelial cells, such as claudin-2, -3, and − 11 [83–85], claudin-1 is more prominent during the developing brain [83, 86] and its sealing function behaves differently from claudin-2 [87, 88]. Furthermore, claudin-1 disruption has been associated with other neurological injuries, such as CNS infection [89], traumatic brain injury [90], and stroke [91], suggesting that this tight junction protein may be involved in the disruption of the ChP-CSF barrier in neonatal PHH. Future studies will evaluate other ChP claudins to determine their association with barrier dysfunction. TEM demonstrated further alterations in cell-to-cell contact after PHH, although clear ultrastructural changes in apical tight junctions were not observed. This may be due to the fact that ZO-1 links to actin [92] and may not be detectable by observations under TEM. Nevertheless, alterations in these tight junctions may cause a dysregulation of the barrier, which may affect the brain integrity and protection from external agents. This may also be related to the influx of immune cell populations into the CSF [93] as a result of the disrupted blood-CSF barrier formed by the ChP epithelium. However, it is also possible that the gaps observed between epithelial cells do not completely disrupt the ChP-CSF barrier. Future studies will include permeability functional tests to assess the extent of barrier disruption and its correlation with tight junction loss after PHH.

Disruption of the ChP-CSF barrier, and consequent immune cell infiltration and inflammation, could contribute to PHH development. There is evidence that ChP-associated inflammation is necessary and sufficient to induce PHH [6, 94]. Inhibition of Toll-like Receptor 4 (TLR4) signaling attenuates ventricular expansion [95], while hyperactivation of inflammation causes ventriculomegaly [96, 97]. Further, administration of steroids reduces ChP-mediated CSF production [98]. In addition to the inflammatory reaction directly mediated by infiltrating immune cells after ChP barrier breakdown, inflamed ChP epithelial cells can increase the expression of TLR4-nuclear factor kappa Β (NF-kΒ) signaling pathways, producing an increase in CSF production and PHH [4, 94].

The ChP has been defined as the site of immune cell transit [99, 100] but also as a reservoir for resident immune cells [32, 43]. Macrophages are innate immune cells that show a variety of immunomodulatory functions including phagocytosis, the release of inflammatory mediators, antigen processing and presentation, and maintenance of immune homeostasis [36]. Macrophages have been divided in three subsets including those that reside in the stromal space [36], those along the apical surface that contact CSF (Kolmer cells) [3], and those who derived from peripheral circulating CD11b^+^ Ly6G^−^ Ly6C^+^ cells [37]. The significant increase of macrophage numbers after intraventricular injection of lysed blood in mouse neonates could be due to the infiltration of circulating CD11b^+^ Ly6G^−^ Ly6C^+^ cells into ChP in response to IVH [65, 101]. However, other mechanisms can be considered, such as proliferation of monocytes/macrophages [4] or migration from other brain areas [102]. It is possible that macrophages are mediating the inflammatory response within the ChP in IVH/PHH and potentially affecting CSF production by the ChP [4, 6]. Notably, macrophage depletion through clodronate-loaded liposomes has attenuated ChP inflammation [103], suggesting ChP macrophages as a therapeutic target in IVH/PHH.

Based on our previous results showing enrichment of immune cells in the CSF of PHH human neonates [93], and research on other conditions showing impaired blood-CSF by ChP macrophages-derived proteases [41], we investigated whether the ChP tight junction impairment may be related to the enrichment of macrophages using ChP explants. While isolated epithelial monolayers are valuable for modeling specific transport kinetics, explant cultures offer a snapshot of the brain’s microenvironment [104]. A primary advantage lies in the preservation of the ChP epithelial cells’ high degree of polarization; in an explant, the apical and basolateral orientations remain intact, whereas 2D cultures often suffer from polarity loss or delayed reorganization [104]. Furthermore, explants provide immediate access to mature tight junctions, such as ZO-1 [105]. Unlike isolated cultures, which require time to reach confluence, explants retain their native barrier strength [105]. Crucially, the ChP explant model preserves the underlying stroma, maintaining the vital interaction between the epithelium and its original tissue architecture.

Peripheral CD11b^+^ Ly6G^−^ Ly6C^+^ cells differentiate to macrophages under inflammatory conditions [106]. Notably, the release of blood products in IVH/PHH-related inflammatory conditions activates immune cells, including macrophages [107]. Our in vitro results show that ZO-1 tight junction disruption occurred when explants were exposed to blood and peripheral CD11b^+^ Ly6G^−^ Ly6C^+^ cells, but not when they were exposed to CD11b^+^ Ly6G^−^ Ly6C^+^ cells or blood alone. Activated macrophages can produce matrix metalloproteinases [108, 109] that can disassemble intercellular junctions and degrade extracellular matrix components [110, 111]. Thus, our in vitro data suggest that the presence of blood may be necessary for the differentiation and/or activation of peripheral CD11b^+^ Ly6G^−^ Ly6C^+^ cells and macrophages. This indicates that blood-activated macrophages might play a role in modulating the integrity of the tight junctions in the ChP as part of the pathophysiology of PHH. The decrease in ZO-1 following exposure to both lysed blood and peripheral CD11b^+^ Ly6G^−^ Ly6C^+^ cells supports an interaction between ZO-1 tight junction disruption, blood and blood products, and macrophages. By contrast, our data suggest that CD11b^+^ Ly6G^−^ Ly6C^+^ cells may not be critically involved in claudin-1 disruption in the context of IVH. Other investigations in hydrocephalus using lipopolysaccharide have also shown downregulation of ZO-1 but not claudins or occludin in the blood-brain barrier [112], suggesting differing responses to stimuli in the proteins that compose a tight junction. ZO-1 is the cytoplasmic scaffolding protein that anchors transmembrane tight junction proteins, including claudin-1, to the actin cytoskeleton and is essential for tight junction organization at the blood-ChP-CSF barrier [43]. When ZO-1 delocalizes in PHH, claudin-1 may remain membrane-associated but become functionally uncoupled, leading to blood-ChP-CSF barrier dysfunction [113]. The differential behavior of claudin-1 between in vitro and in vivo models may be related to timing (7 days vs. 24 h post-blood exposure). Temporal dynamics of protein cleavage may vary; specifically, claudin-1 may be more resistant to degradation than ZO-1. The 48-h viability threshold of our ChP explant culture system precludes longer-term observations. Future studies will elucidate the molecular mechanisms by which blood activates peripheral CD11b^+^ Ly6G^−^ Ly6C^+^ cells and macrophages and their association with tight junction disruption in PHH.

Our study had several limitations. Our mouse model displayed some variability in the development of PHH and ventricular size; however, this wide distribution in ventricle sizes is a pattern that is consistent with the clinical course of IVH/PHH and other experimental models [74]. Bilateral injections may cause more tissue damage than unilateral injections, particularly mechanical injury along the needle track in the neocortex. However, the absence of hydrocephalus in the sham controls and our quantifications in the neocortex suggest that the procedure did not produce neocortical damage and effectively induced PHH. Long-term changes were not examined and functional outcomes were not assessed in this investigation, but future studies will include them. Similarly, both sexes were included in this study but differences were not evaluated; future studies will increase the sample size to study sex differences. The in vitro study allowed us to focus on the action of CD11b^+^ Ly6G^−^ Ly6C^+^ cells in ChP cell junctions under controlled confounding variables; however, this type of experiments does not provide a complete view of the complex living organism [114]. Future experiments will be designed to uncover the role of CD11b^+^ Ly6G^−^ Ly6C^+^ cell-derived macrophages in vivo and their correlation with disrupted ChP junctions. Based on literature [35, 115], we used the CD206 marker and location to differentiate macrophages from microglia but could not differentiate between resident and infiltrating macrophages in the ChP. While the sorted CD11b^+^ Ly6G^−^ Ly6C^+^ cells demonstrated 99.1% purity, it remains possible that CD11b^+^ F4/80^+^ macrophages may also be present. Future studies will focus on increasing the markers in our flow cytometry panel to determine the source of macrophages more accurately in PHH pathophysiology. Finally, we acknowledge the heterogeneity (e.g., estimated gestational fetal age, comorbidities, IVH grade, sample size, no possibility of TEM analyses) inherent to such studies in rare human post-mortem samples.

## Conclusions

Our study showed ChP epithelial disruption with a decrease in tight junction proteins of the epithelial cells and associated enrichment of activated macrophages in PHH. In vitro studies demonstrated the dependency of CD11b^+^ Ly6G^−^ Ly6C^+^ cells and lysed blood for ChP tight junction disruption. As there are currently no non-surgical treatments for PHH, our results have the potential to shape immunomodulatory therapies for PHH and improve the lives of children affected by this condition.

## Supplementary Information

Below is the link to the electronic supplementary material.

Supplementary Material 1: Fig. 1. Characterization of the neonatal mouse model of PHH. (a) MR images of a representative control and PHH mice after 30 days post-induction. Behavioral tests performed at P6 and P8: (b) surface righting, (c) negative geotaxis, and (d) forced swim. (e) Representative image of neocortical layers II-III and layer VI using NeuN + DAPI staining. (f) Number of total DAPI+ cells per 10,000 µm2 and divided in layers II-III (g), and VI (h). Micrographs of NeuN immunostaining in layers II-III and VI in (I, k) mouse sham controls and mouse PHH (j, l), respectively. Dot plots showing the percentage of (m) total NeuN+ cells in the neocortex and in (n) layers II-III and (o) layer VI. Representative images of cleaved caspase 3 and NeuN immunofluorescences in (p) a mouse sham control and (q) a PHH mouse. (r) Detail of the cell pointed with a white arrow in q. (s) Detail of a NeuN+ cells co-labelled with cleaved caspase 3. Separate channels are shown in r`-r``` and s`’s``` for the cells in r and s, respectively. Note the co-localization of DAPI, NeuN and cleaved caspase 3 in s-s``` but not in r-r```. Dot plots showing the percentage of (t) total cleaved caspase 3+ cells without NeuN and (u) co-localizing with NeuN. N = 7 sham controls and n = 8–11 PHH mice were used. Means ± SD are shown. Two-tailed Wilcoxon–Mann–Whitney test was applied.

Supplementary Material 2: Fig. 2. Unstained controls. Gating strategy showing a representative unstained control including < 0.1% autofluorescent cells used for spectral flow cytometry.

Supplementary Material 3: Fig. 3. Correlation between ventricular volume and immunofluorescent markers in mouse PHH. No correlation was found between ventricular volume and (a) Iba1^+^ cells, (b) iba1^+^ CD68^+^ cells, (c) ZO-1, or (d) Claudin-1, in PHH (*n* = 7), (e-h) and amongst those markers (*n* = 11). See e for ZO-1 vs. iba1^+^ cells, f for claudin-1 vs. iba1^+^ cells, g for ZO-1 vs. iba1^+^CD68^+^ cells, and h for claudin-1 vs. iba1^+^CD68^+^ cells. Simple linear regression was used to calculate R^2^ values and *p*-values (see in graphs).

Supplementary Material 4: Fig. 4. An in vitro model of mouse ChP. (a) Diagram showing in vitro studies of ChP. Immunofluorescence of in vitro ChP tight junction protein of (b) claudin-1 (fluorescence in *green*) and βIV tubulin (*red*), and (c) aquaporin-1 (*green*). Separate channels are shown in b`, b`` and c`. Images were obtained under a fluorescent microscope. DAPI stained all nuclei in blue. Abbreviations: *ChP* choroid plexus.

Supplementary Material 5: Fig. 5. Gating strategy for sorting splenic monocytes from mice.

Supplementary Material 6: Fig. 6. Gating strategy of splenic monocytes post-sorting from mice.

Supplementary Material 7: Table 1. Human infant post-mortem cases.

Supplementary Material 8: Table 2. Primary antibodies.

Please use this file to replace Supplementary Fig. 1. There was an error in the title of graph (n). The caption is: Supplementary Material 1: Fig. 1. Characterization of the neonatal mouse model of PHH. (a) MR images of a representative control and PHH mice after 30 days post-induction. Behavioral tests performed at P6 and P8: (b) surface righting, (c) negative geotaxis, and (d) forced swim. (e) Representative image of neocortical layers II-III and layer VI using NeuN + DAPI staining. (f) Number of total DAPI+ cells per 10,000 µm2 and divided in layers II-III (g), and VI (h). Micrographs of NeuN immunostaining in layers II-III and VI in (I, k) mouse sham controls and mouse PHH (j, l), respectively. Dot plots showing the percentage of (m) total NeuN+ cells in the neocortex and in (n) layers II-III and (o) layer VI. Representative images of cleaved caspase 3 and NeuN immunofluorescences in (p) a mouse sham control and (q) a PHH mouse. (r) Detail of the cell pointed with a white arrow in q. (s) Detail of a NeuN+ cells co-labelled with cleaved caspase 3. Separate channels are shown in r`-r``` and s`’s``` for the cells in r and s, respectively. Note the co-localization of DAPI, NeuN and cleaved caspase 3 in s-s``` but not in r-r```. Dot plots showing the percentage of (t) total cleaved caspase 3+ cells without NeuN and (u) co-localizing with NeuN. N = 7 sham controls and n = 8–11 PHH mice were used. Means ± SD are shown. Two-tailed Wilcoxon–Mann–Whitney test was applied.

## Data Availability

All data generated or analyzed during this study are included in this published article [and its supplementary information files]. Any additional information reported in this paper is available from the corresponding author on reasonable request.

## Abbreviations

ChP: Choroid plexus
CSF: Cerebrospinal fluid
IVH: Intraventricular hemorrhage
MRI: Magnetic resonance imaging
PHH: Post-hemorrhagic hydrocephalus
SD: Standard deviation
TEM: Transmission electron microscopy
