# Supplementary figures and images for: Post-hemorrhagic hydrocephalus of prematurity is associated with disruption of tight junctions and increased macrophage activity in the choroid plexus
Source: Fluids Barriers CNS. 2026 Mar 31;23:73. doi: 10.1186/s12987-026-00800-x (PMC13170321; doi:10.1186/s12987-026-00800-x)

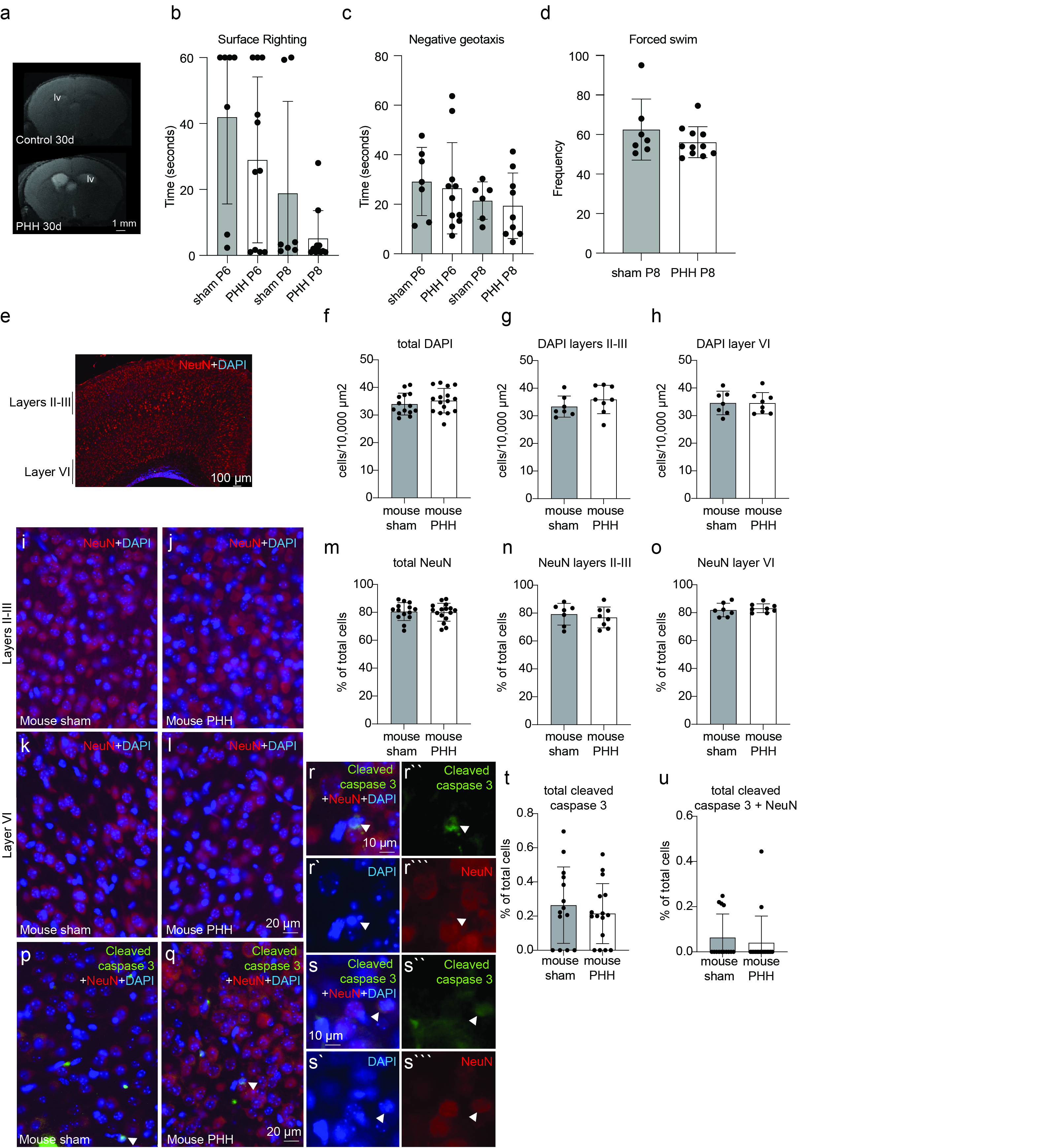

Supplement: Supplementary file 1 — Supplementary Material 1: Fig. 1. Characterization of the neonatal mouse model of PHH. (a) MR images of a representative control and PHH mice after 30 days post-induction. Behavioral tests performed at P6 and P8: (b) surface righting, (c) negative geotaxis, and (d) forced swim. (e) Representative image of neocortical layers II-III and layer VI using NeuN + DAPI staining. (f) Number of total DAPI+ cells per 10,000 µm2 and divided in layers II-III (g), and VI (h). Micrographs of NeuN immunostaining in layers II-III and VI in (I, k) mouse sham controls and mouse PHH (j, l), respectively. Dot plots showing the percentage of (m) total NeuN+ cells in the neocortex and in (n) layers II-III and (o) layer VI. Representative images of cleaved caspase 3 and NeuN immunofluorescences in (p) a mouse sham control and (q) a PHH mouse. (r) Detail of the cell pointed with a white arrow in q. (s) Detail of a NeuN+ cells co-labelled with cleaved caspase 3. Separate channels are shown in r`-r``` and s`’s``` for the cells in r and s, respectively. Note the co-localization of DAPI, NeuN and cleaved caspase 3 in s-s``` but not in r-r```. Dot plots showing the percentage of (t) total cleaved caspase 3+ cells without NeuN and (u) co-localizing with NeuN. N = 7 sham controls and n = 8–11 PHH mice were used. Means ± SD are shown. Two-tailed Wilcoxon–Mann–Whitney test was applied. [file 12987_2026_800_MOESM1_ESM.tif]

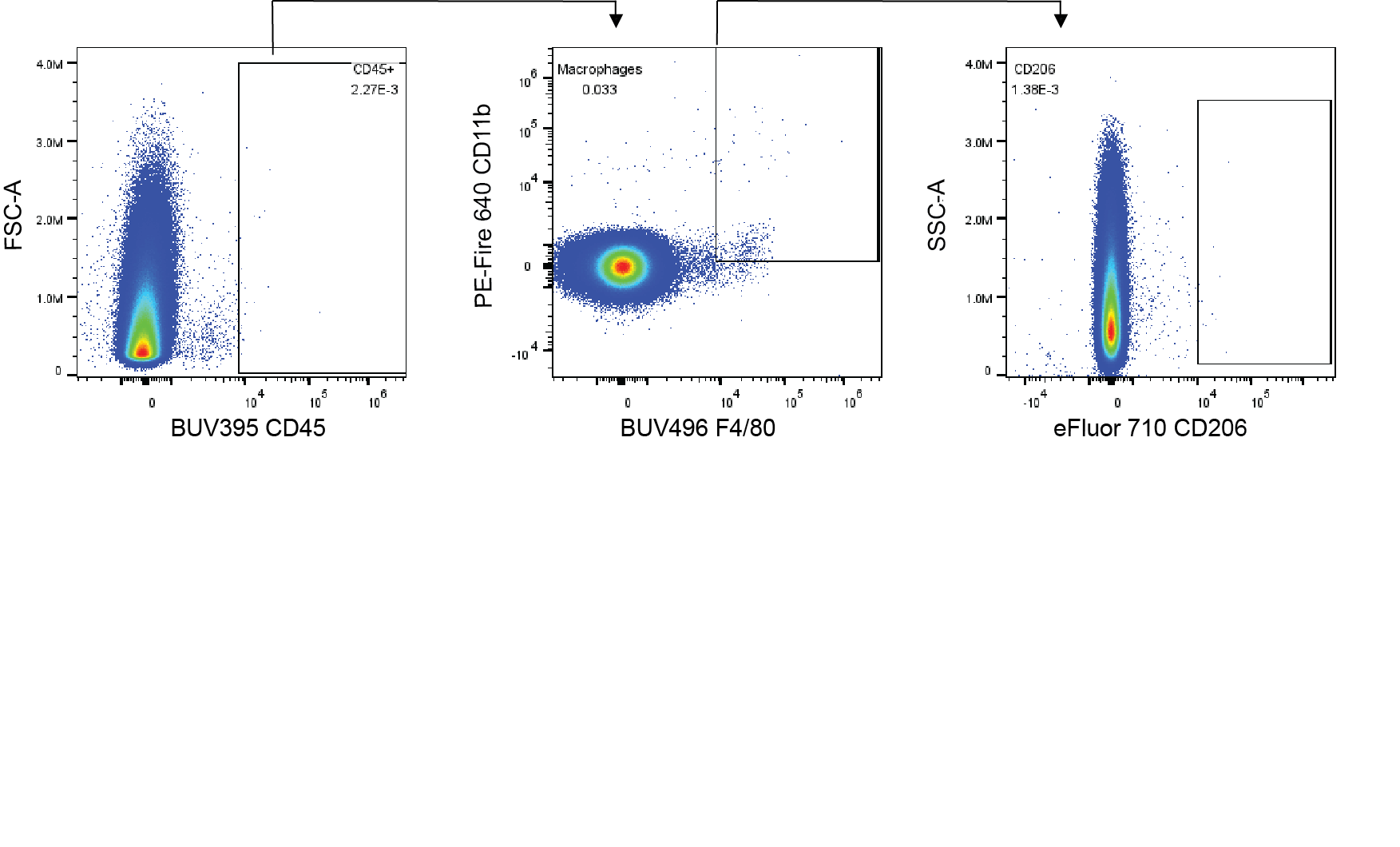

Supplement: Supplementary file 2 — Supplementary Material 2: Fig. 2. Unstained controls. Gating strategy showing a representative unstained control including < 0.1% autofluorescent cells used for spectral flow cytometry. [file 12987_2026_800_MOESM2_ESM.tiff]

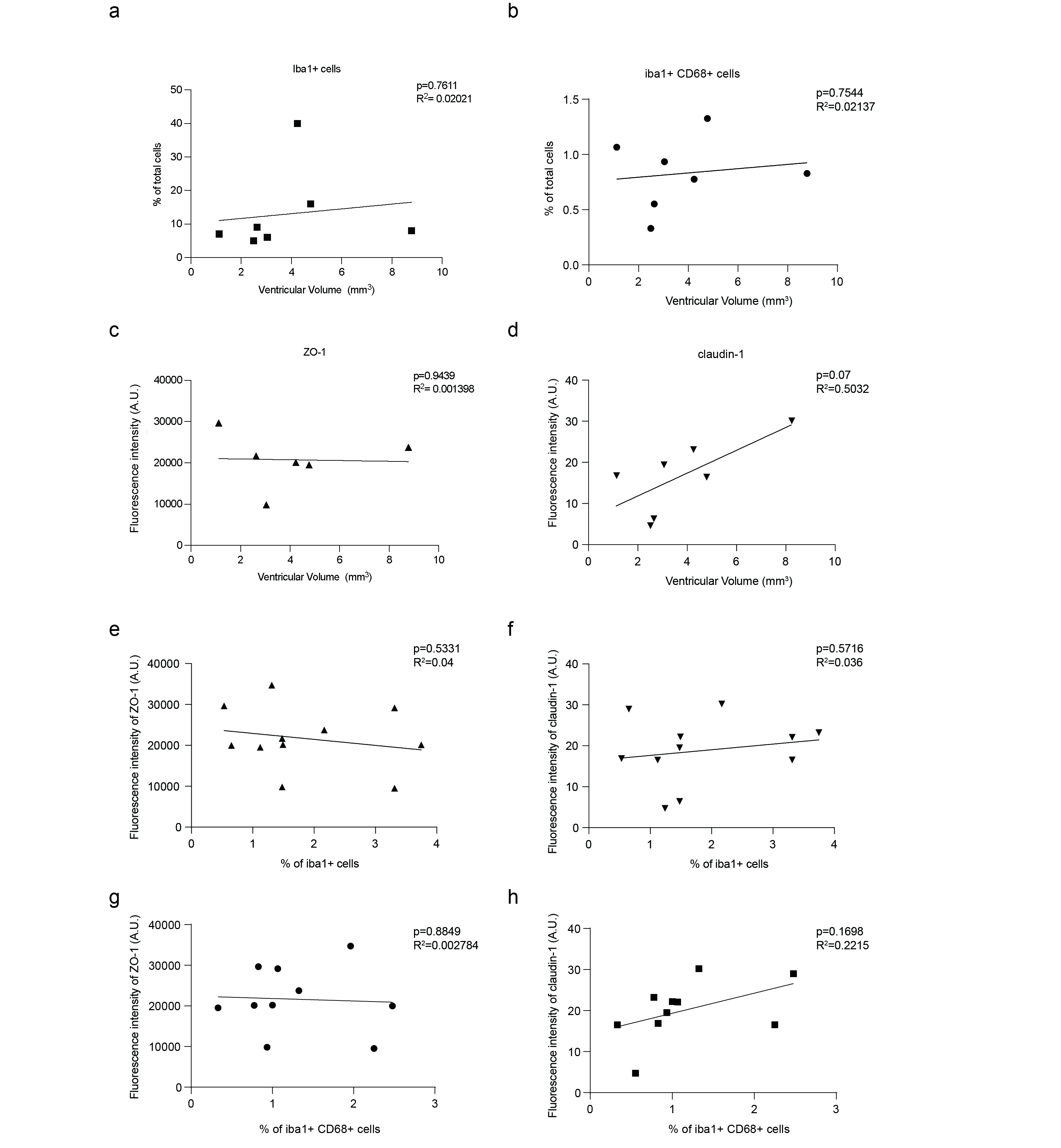

Supplement: Supplementary file 3 — Supplementary Material 3: Fig. 3. Correlation between ventricular volume and immunofluorescent markers in mouse PHH. No correlation was found between ventricular volume and (a) Iba1+ cells, (b) iba1+ CD68+ cells, (c) ZO-1, or (d) Claudin-1, in PHH (n = 7), (e-h) and amongst those markers (n = 11). See e for ZO-1 vs. iba1+ cells, f for claudin-1 vs. iba1+ cells, g for ZO-1 vs. iba1+CD68+ cells, and h for claudin-1 vs. iba1+CD68+ cells. Simple linear regression was used to calculate R2 values and p-values (see in graphs). [file 12987_2026_800_MOESM3_ESM.tiff]

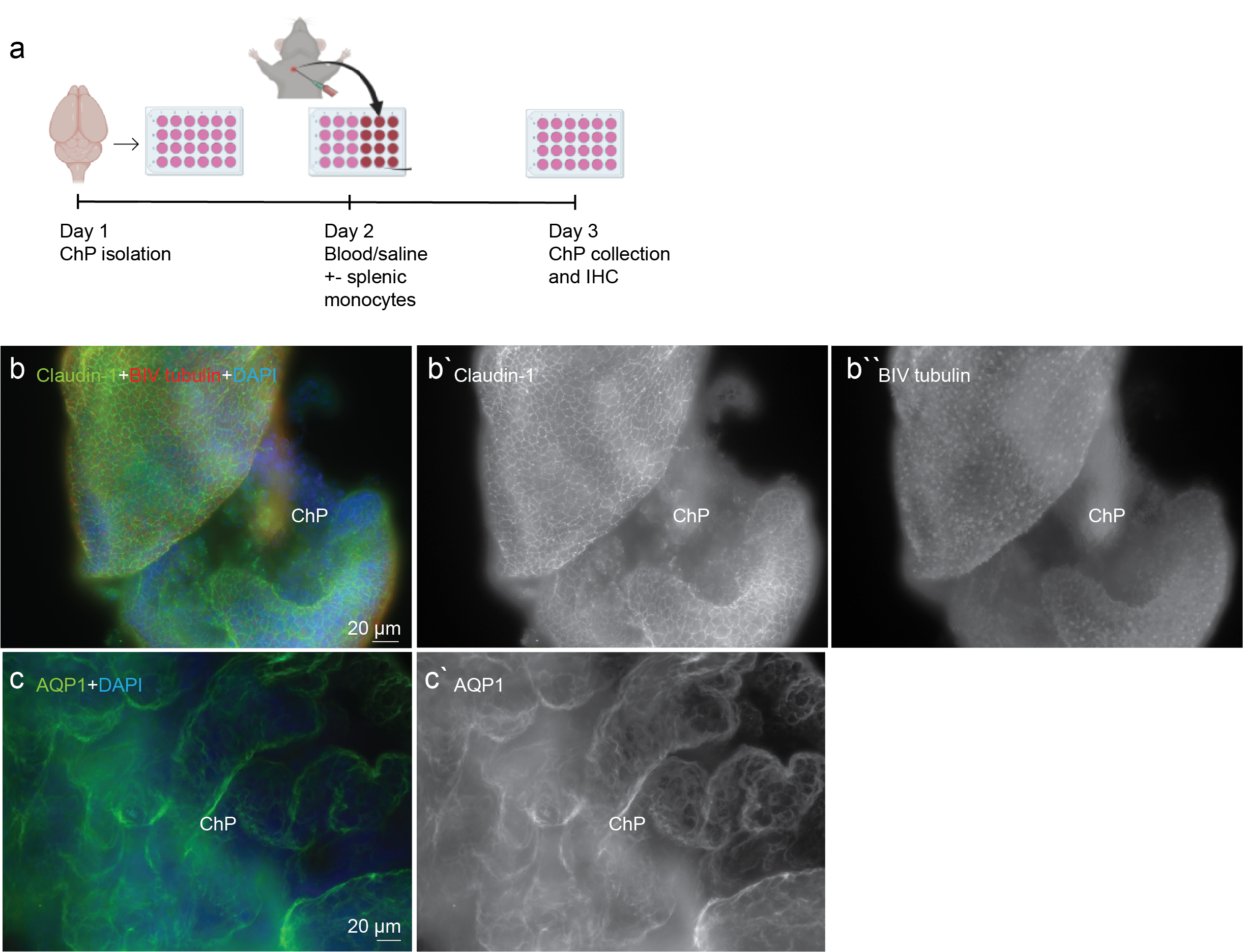

Supplement: Supplementary file 4 — Supplementary Material 4: Fig. 4. An in vitro model of mouse ChP. (a) Diagram showing in vitro studies of ChP. Immunofluorescence of in vitro ChP tight junction protein of (b) claudin-1 (fluorescence in green) and βIV tubulin (red), and (c) aquaporin-1 (green). Separate channels are shown in b`, b`` and c`. Images were obtained under a fluorescent microscope. DAPI stained all nuclei in blue. Abbreviations: ChP choroid plexus. [file 12987_2026_800_MOESM4_ESM.tiff]

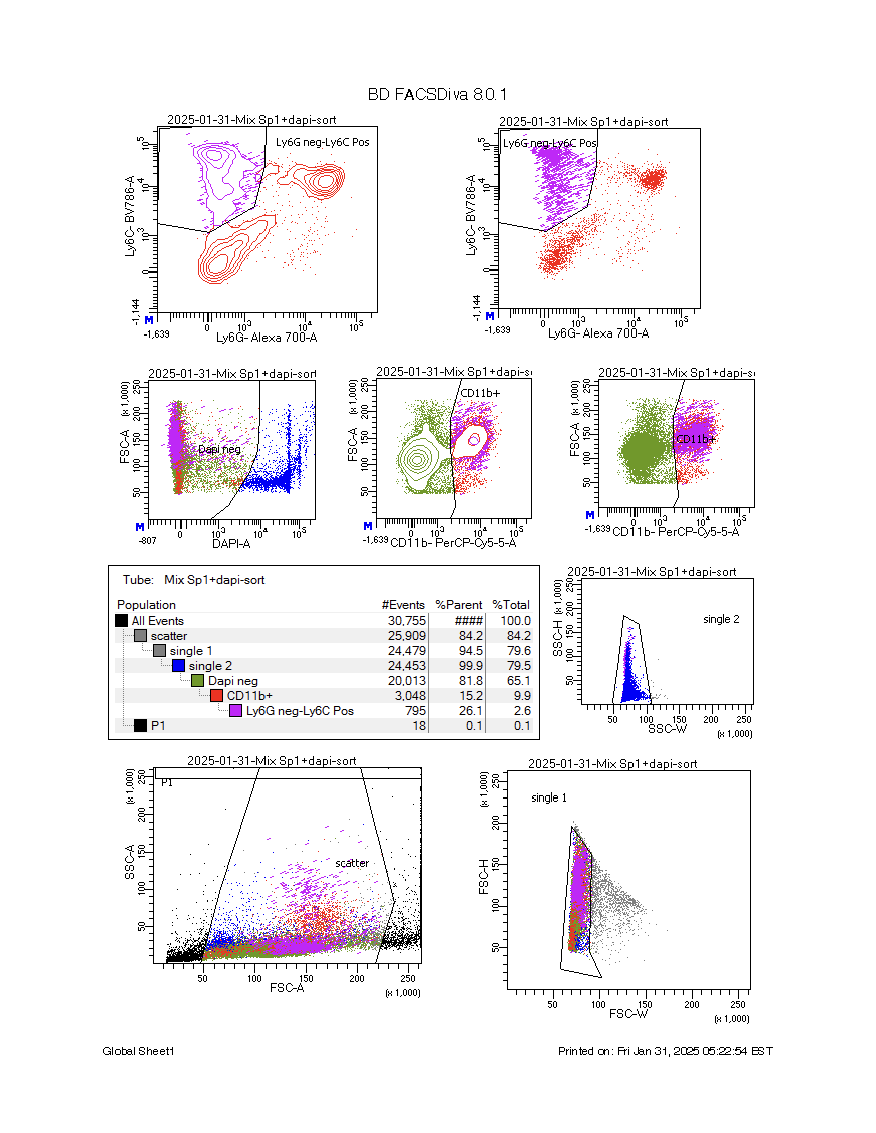

Supplement: Supplementary file 5 — Supplementary Material 5: Fig. 5. Gating strategy for sorting splenic monocytes from mice. [file 12987_2026_800_MOESM5_ESM.tiff]

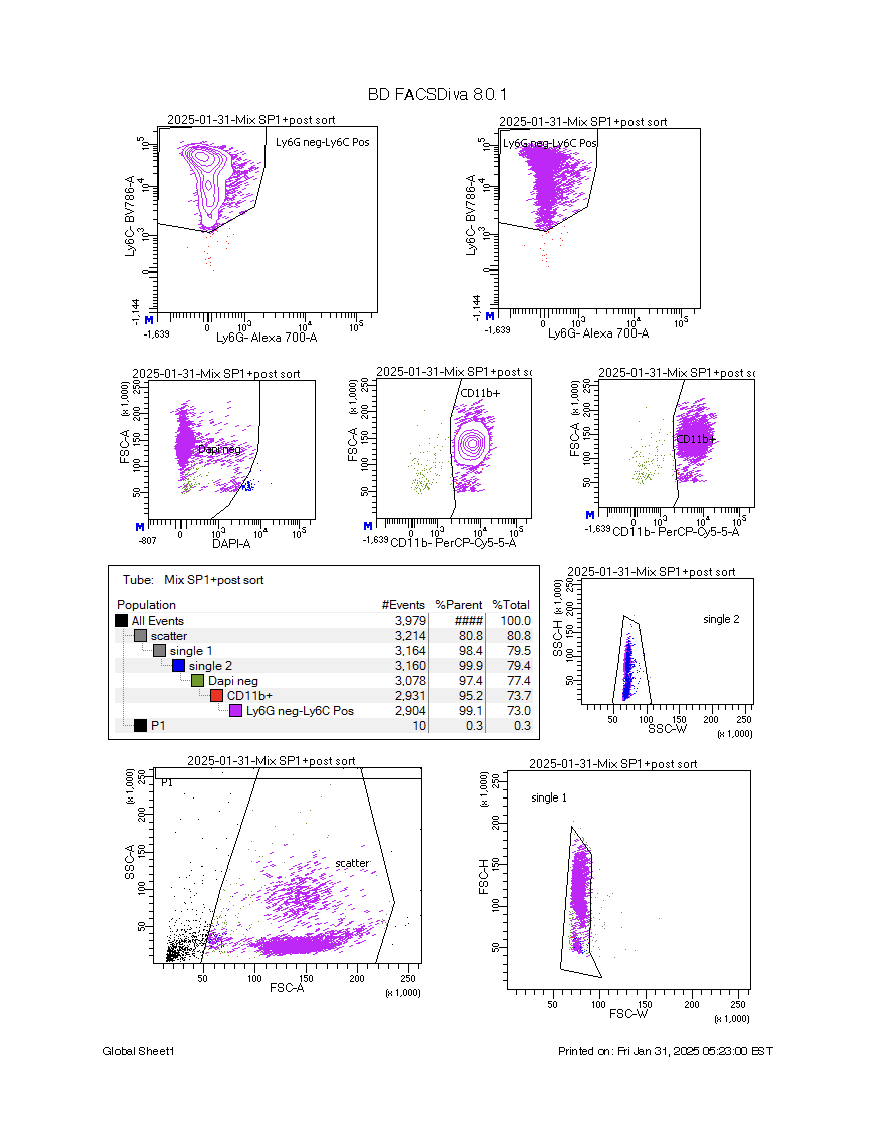

Supplement: Supplementary file 6 — Supplementary Material 6: Fig. 6. Gating strategy of splenic monocytes post-sorting from mice. [file 12987_2026_800_MOESM6_ESM.tiff]
