# Supplementary material for: Post-hemorrhagic hydrocephalus of prematurity is associated with disruption of tight junctions and increased macrophage activity in the choroid plexus
Source: Fluids Barriers CNS. 2026 Mar 31;23:73. doi: 10.1186/s12987-026-00800-x (PMC13170321; doi:10.1186/s12987-026-00800-x)
Supplement: Supplementary file 7 — Supplementary Material 7: Table 1. Human infant post-mortem cases. [file 12987_2026_800_MOESM7_ESM.docx]

| Supplemental Table 1. Human infant postmortem cases. | | | | |
| --- | --- | --- | --- | --- |
| Condition | **Gender** | **Ethnicity** | **EGFA birth (weeks+days)** | **Pathology and imaging notes** |
| Intracranial control | M | African American | 23+6d | No IVH or PHH pathology. |
| Intracranial control | M | African American | 26+5d | No IVH or PHH pathology. |
| Intracranial control | M | African American | 23+6d | No IVH or PHH pathology. |
| IVH Grade 1 PVL | M | Unknown | 22 | Right germinal matrix/subependymal hemorrhage. R>L possible periventricular leukomalacia. |
| IVH Grade 3-4 | M | Unknown | 27 | Subependymal hemorrhage, ventricles dilated with 2x1x2.6cm hematoma in the lateral ventricles. Posterior fossa epidural hematoma. |
| IVH Grade 4 | M | African American | 28+3d | Large intraparenchymal hemorrhage with intraventricular extension - grade 4. Ventricles dilated. |
| Abbreviations: EGFA, estimated gestational fetal age; IVH, intraventricular hemorrhage; PVL, periventricular leukomalacia. Control cases showed other non-intracranial systemic pathologies, including intrauterine growth restriction, respiratory distress syndrome, or pulmonary hemorrhage. | | | | |
