# Supplementary material for: Post-hemorrhagic hydrocephalus of prematurity is associated with disruption of tight junctions and increased macrophage activity in the choroid plexus
Source: Fluids Barriers CNS. 2026 Mar 31;23:73. doi: 10.1186/s12987-026-00800-x (PMC13170321; doi:10.1186/s12987-026-00800-x)
Supplement: Supplementary file 8 — Supplementary Material 8: Table 2. Primary antibodies. [file 12987_2026_800_MOESM8_ESM.docx]

| **Supplemental Table 2.** Primary antibodies. | | | | | | | |
| --- | --- | --- | --- | --- | --- | --- | --- |
| **Antibody** | **Target** | **Manufacturer** | **Catalogue number** | **Research Resource Identifier** | **Dilution** | **Host** | **Experiment (Fluorophore)** |
| AQP1 | Water channel | Abnova (Taiwan, China) | PAB27167 | N/A | 1:500 | Rabbit | IF |
| βIV tubulin | Cilia | Abcam | Ab11315 | AB_297919 | 1:50 | Mouse | IF |
| CD11b | Microglia/Macrophages | BioLegend | 101280  101227 | AB_2888802  AB_893233 | 0.2 mg/ml  1:1000 | Rat | FC (PE/Fire640)  FC (PerCP/Cyanine5.5) |
| CD206 | Macrophages | eBiosciences | 46-2061-82 | AB_2784688 | 0.125 µg/test | Rat | FC (PerCP-eFluor 710) |
| CD45 | Immune cells | BD Bioscience | Bdb564279 | AB_2651134 | 0.2 mg/ml | Rat | FC (BUV395) |
| CD68 | Lysosomes/endosomes | Abcam | ab125212 | AB_10975465 | 1:500 | Rabbit | IF |
| Claudin-1 | TJs in ChP epithelium | Invitrogen | 71-7800 | AB_88416 | 1:25 | Rabbit | IF |
| Cleaved caspase 3 | Cell death | Cell Signaling Technology | 9661T | AB_2341188 | 1:400 | Rabbit | IF |
| F4/80 | Macrophages | BD Bioscience | Bdb750644 | AB_2874772 | 0.2 mg/ml | Rat | FC (BUV496) |
| Iba1 | Microglia/Macrophages | ThermoFisher Scientific  Abcam | NC9288364  Ab5076 | AB_839504  AB_2224402 | 1:500  1:100 | Rabbit  Goat | IF |
| Ly6C | Monocytes | BioLegend | 128023 | AB_10640119 | 1:1000 | Rat | FC (Alexa 700) |
| Ly6G | Granulocytes | BioLegend | 127645 | AB_2566317 | 1:1000 | Rat | FC (BV785) |
| NeuN | Neurons | Sigma-Aldrich | MAB377A5 | AB_2814948 | 1:500 | Mouse | IF |
| ZO-1 | TJs in ChP epithelium | Invitrogen | MA5-46951 | AB_2938023 | 1:25 | Rabbit | IF |
| Abbreviations: FC, flow cytometry; IF, immunofluorescence; N/A, not available. | | | | | | | |
